# *Ppia* is the most stable housekeeping gene for qRT-PCR normalization in kidneys of three *Pkd1*-deficient mouse models

**DOI:** 10.1038/s41598-021-99366-x

**Published:** 2021-10-05

**Authors:** Juan J. Muñoz, Ana C. Anauate, Andressa G. Amaral, Frederico M. Ferreira, Elieser H. Watanabe, Renata Meca, Milene S. Ormanji, Mirian A. Boim, Luiz F. Onuchic, Ita P. Heilberg

**Affiliations:** 1 Nephrology Division, Department of Medicine, Universidade Federal de São Paulo, Rua Botucatu 740 - Vila Clementino, São Paulo, 04023-900 Brazil; 2 Divisions of Molecular Medicine and Nephrology, University of São Paulo School of Medicine, São Paulo, Brazil; 3 Division of Pathology, University of São Paulo School of Medicine, São Paulo, Brazil

**Keywords:** Computational biology and bioinformatics, Genetics, Molecular biology, Biomarkers, Diseases, Nephrology

## Abstract

Autosomal Dominant Polycystic Kidney Disease (ADPKD) is the most common inherited renal disorder, characterized by renal cyst development leading to end-stage renal disease. Although the appropriate choice of suitable reference is critical for quantitative RNA analysis, no comparison of frequently used “housekeeping” genes is available. Here, we determined the validity of 7 candidate housekeeping genes (*Actb*, *Actg1*, *B2m*, *Gapdh*, *Hprt*, *Pgam1* and *Ppia*) in kidney tissues from mouse models orthologous to ADPKD, including a cystic mice (CY) 10–12 weeks old (*Pkd1*^flox/flox^:*Nestin*^cre^/*Pkd1*^flox/−^:*Nestin*^cre^, n = 10) and non-cystic (NC) controls (*Pkd1*^flox^/^flox^/*Pkd1*^flox^/^-^, n = 10), *Pkd1*-haploinsufficient (HT) mice (*Pkd1*^+/−^, n = 6) and wild-type (WT) controls (*Pkd1*^+/+^, n = 6) and a severely cystic (SC) mice 15 days old (*Pkd1*^V/V^, n = 7) and their controls (CO, n = 5). Gene expression data were analyzed using six distinct statistical softwares. The estimation of the ideal number of genes suggested the use of *Ppia* alone as sufficient, although not ideal, to analyze groups altogether. *Actb*, *Hprt* and *Ppia* expression profiles were correlated in all samples. *Ppia* was identified as the most stable housekeeping gene, while *Gapdh* was the least stable for all kidney samples. *Stat3* expression level was consistent with upregulation in SC compared to CO when normalized by *Ppia* expression. In conclusion, present findings identified *Ppia* as the best housekeeping gene for CY + NC and SC + CO groups, while *Hprt* was the best for the HT + WT group.

## Introduction

Autosomal dominant polycystic kidney disease (ADPKD) is manifested by bilateral development of multiple fluid-filled epithelial-derived cysts^1,2^. It is the most common Mendelian disorder of the kidneys, affecting 3–5:10,000 people worldwide^3^ and the leading monogenic cause of end-stage kidney disease^4^. Mutations in the *PKD1* gene account for ~ 78% of the affected families while mutations in *PKD2* are detected in ~ 15% of them^5,6^, with the remaining ones being genetically unresolved or associated with rare mutations in the *GANAB* and *DNAJB11* loci^7^.

The elucidation of *PKD1*/*PKD2*-related biology has allowed major steps toward the understanding of ADPKD pathogenesis and the development of diagnostic tools and biomarkers^8^. The significant number of abnormally functioning pathways involved in the disease pathophysiology, in turn, opened a number of tracks to develop potentially specific therapies. In addition to eGFR, the development of progression and prognostic markers has been also essential to guide clinical decisions in ADPKD. The classification based on total kidney volume growth^9^ has been widely used as well as the multiple criteria developed by Cornec-Le Gal et al.^10^ A combination of distinct biomarkers in a classifier including new biomarkers should further increase sensitivity and specificity^11^. The characterization and elucidation of specific strategic transcriptional profiles in turn, are expected to expand the options of therapeutic targets and the number of useful biomarkers for the disease.

Gene expression analysis plays a central role on identifying and characterizing pathways involved in specific phenotypes and diseases, potentially allowing elucidation of pathogenetic aspects and biomarker discovery. Reverse transcription quantitative polymerase chain reaction (RT-qPCR) is one of the most sensitive and reproducible means of quantifying RNA expression^12,13^. Nonetheless, to provide accurate expression analysis this method requires expression normalization of the genes of interest to a reference gene that is stable and not affected by experimental conditions. In this scenario, expression stability is a major criterion for housekeeping gene selection^14^. Reference genes are generally selected among housekeeping genes ubiquitously expressed and not transcriptionally affected by experimental conditions^13^. While the expression of some housekeeping genes is constant under certain conditions, it can significantly change in some circumstances^15^ such as developmental stages, cell types and experimental conditions^13,14,16,17^. It is therefore essential to characterize the suitability of candidate housekeeping genes to serve as appropriate internal mRNA expression controls under a given experimental condition where transcriptional effects are being investigated.

Glyceraldehyde-3-phosphate dehydrogenase (*Gapdh*), actin beta (*Actb*) and 18 s ribosomal RNA (*18 s rRNA*) are the most common reference genes. In kidney tissues of *Pkd1*-deficient mouse models, *Gapdh* has been the most often used endogenous control for gene expression studies^18^. However, its reliability in this context has not been demonstrated yet. Moreover, the indiscriminate use of these genes may be inappropriate, as they have been implicated in disease processes^19^ including ADPKD^20^. Therefore, whether the expression of housekeeping genes is or is not stable in kidney tissues of *Pkd1*-deficient mouse models remains unknown. Determining the stability of genes known to be involved in ADPKD and used as housekeeping is particularly important.

To elucidate this issue and bring appropriate information to PKD experimental studies involving kidney transcriptional profiles, we aimed to identify the most stable housekeeping controls from a panel, namely seven candidate genes commonly used as endogenous controls in kidney disease, not limited to ADPKD: *Actb*, *Actg1*, *B2m*, *Gapdh*, *Hprt*, *Pgam1*, and *Ppia*., to be considered in kidney RT-qPCR obtained from *Pkd1*-deficient mouse models.

## Results

### Quantitative gene expression analyses: the first step to identify optimal housekeeping genes for RT-qPCR studies of renal tissue in *Pkd1*-deficient mouse models

**Figure 1:**
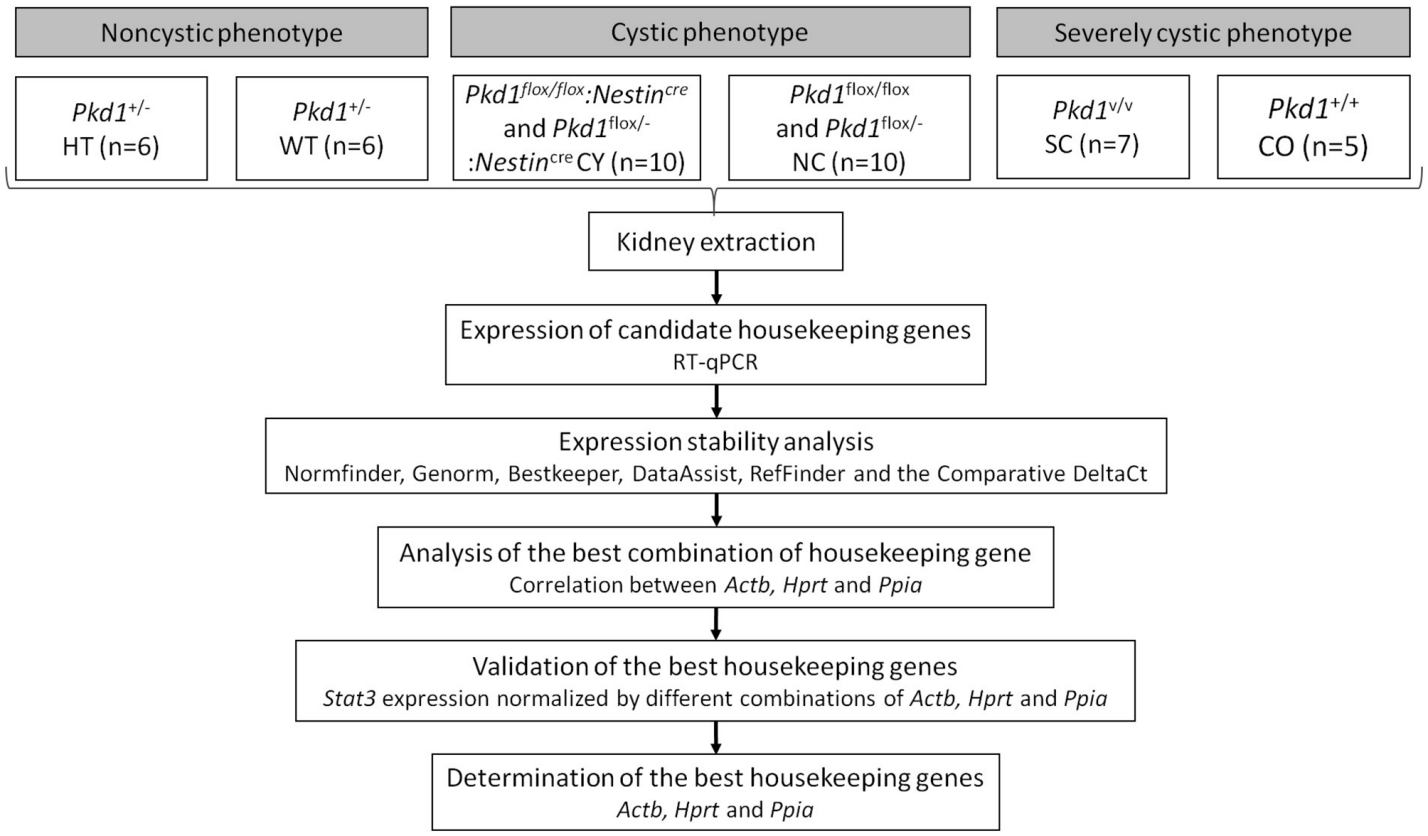
Workflow diagram illustrating the strategy used to identify the best housekeeping normalizer for RT-qPCR studies in *Pkd1*-deficient kidneys. To proceed with appropriate comparisons, we included housekeeping genes selected from the available literature (*Actb*, *Actg1*, *B2m*, *Gapdh*, *Hprt*, *Pgam1* and *Ppia*). *Pkd1*, polycystic kidney disease 1 gene.

We applied a stepwise strategy to select and evaluate candidate housekeeping genes with the aim of identifying optimal housekeeping genes for RT-qPCR analyses of kidney tissue in three *Pkd1*-deficient mouse models: *Pkd1*^+/−^ [haploinsufficient (HT), noncystic] and its control *Pkd1*^+/+^ (wild type, WT); *Pkd1*^flox/flox^:*Nestin*^cre^ and *Pkd1*^flox/−^:*Nestin*^cre^ (cystic, CY) and their controls *Pkd1*^flox/flox^ and *Pkd1*^flox/−^ (noncystic, NC); and *Pkd1*^V/V^ [severely cystic, SC) and its control *Pkd1*^+/+^ (CO, noncystic). Such steps are outlined in Fig. 1.

**Figure 2:**
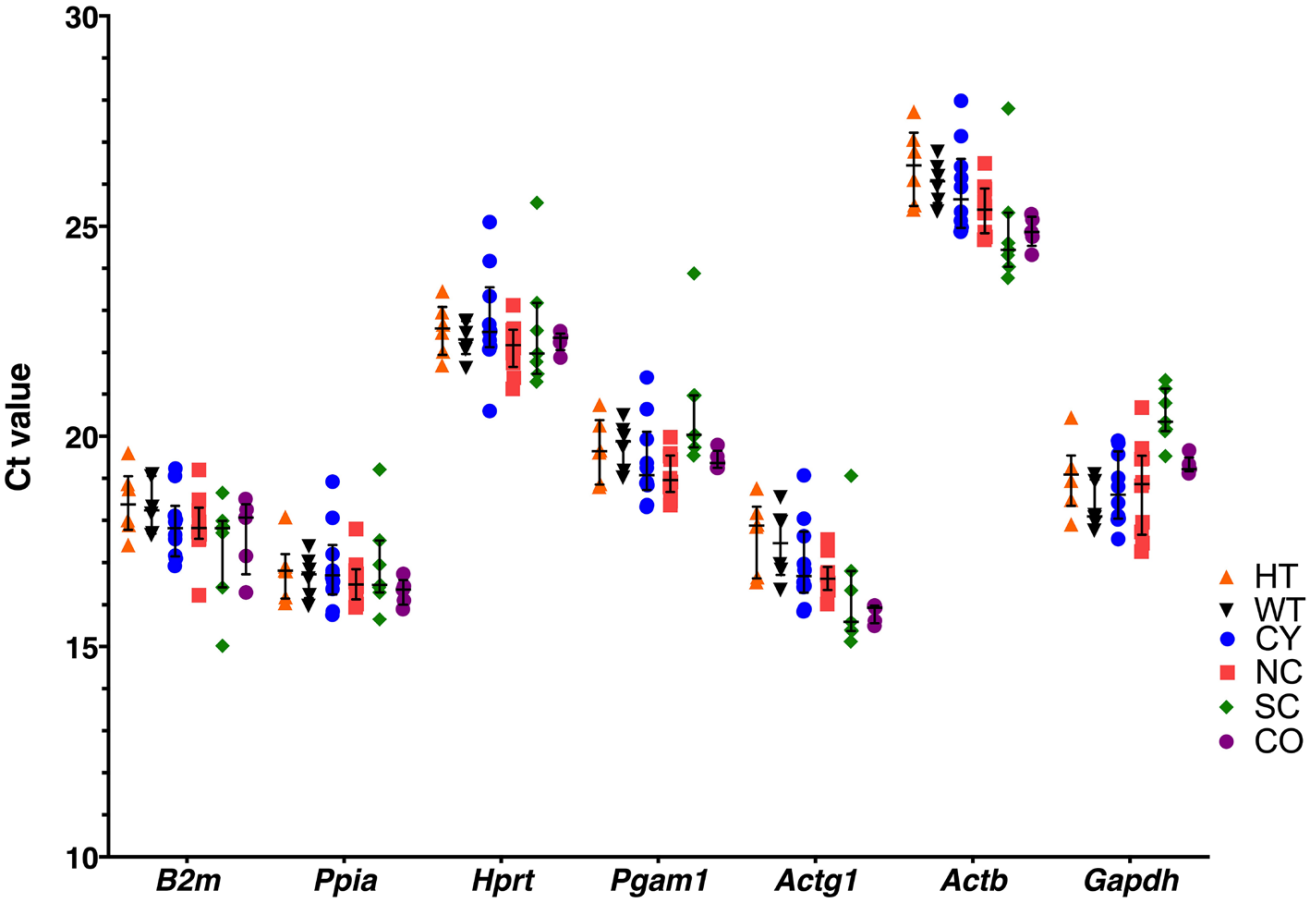
Renal expression profiles of the seven selected candidate housekeeping genes in *Pkd1*-deficient mouse models. Lower threshold values (Ct) indicate higher expression levels. The median values are expressed as horizontal lines while the error bars represent the corresponding interquartile ranges. The *Actb* Ct values were the highest ones, reflecting the lowest expression levels whereas the *Actg1* Ct values were the lowest, indicating the highest expression.

Raw RT-qPCR data in renal tissue were obtained for the *Actb*, *Actg1*, *B2m*, *Gapdh*, *Hprt*, *Pgam1* and *Ppia* candidate housekeeping genes for each of the *Pkd1*-deficient mouse models. The corresponding median Ct values are shown in Fig. 2. High expression levels were obtained for *Actg1* [16.60 (1.61)], *B2m* [19.94 (0.88)], *Gapdh* [19.11 (1.63)], *Pgam1* [19.52 (1.10)] and *Ppia* [16.60 (0.75)], with median Cts lying between 15 and 20. *Hprt* [22.37 (0.69)] and *Actb* [25.38 (1.32)], in turn, displayed moderate (Ct between 20 and 25) and relatively low expression (Ct above 25), respectively (Fig. 2). This process was preceded by assessment of RNA quality, which showed that RIN (RNA Integration Numbers) ranged from 5.9 to 8.8 among the analyzed samples (Supplementary Table S4).

Separate assessments and comparisons involving the renal cystic mice and their corresponding controls were performed, including only the *Pkd1*^flox/flox^:*Nestin*^cre^ and *Pkd1*^flox^/^flox^ genotypes as well as both cystic genotypes and corresponding controls: *Pkd1*^flox/flox^:*Nestin*^cre^, *Pkd1*^flox/−^:*Nestin*^cre^, *Pkd1*^flox/flox^ and *Pkd1*^flox^/^-^ (Supplementary Tables S2 and S3). The results yielded by the two analyses did not significantly differ, showing similarity and stability of genetic background between both model pairs. Of note, this pattern was also observed between such model pairs in a previous study of ours directed to identifying housekeeping genes for microRNA expression analysis^21^. In this context, in the current study we analyzed this cystic model merging *Pkd1*^flox/flox^:*Nestin*^cre^ and *Pkd1*^flox/−^:*Nestin*^cre^ mice in the CY group and *Pkd1*^flox/flox^ and *Pkd1*^flox^/^-^ in the NC group.

### Analysis of expression stability of the candidate housekeeping genes

The following step in the process of selecting the best housekeeping genes was the assessment of expression stability for each of the candidates, taking into account the related genetic backgrounds. This task was accomplished using the NormFinder, GeNorm, RefFinder, ΔCt method, Bestkeeper and DataAssist software packages (Fig. 2), which allowed determining their relative expression stability and generating a ranking among the best ones. The most stably expressed genes in the kidney samples of the three *Pkd1*-deficient mouse models were defined by the lowest variability values observed in each condition of interest. It must be pointed out that the results for the CY-NC model did not differ between the two and four-genotype analyses (Supplementary Tables S2 and S3).

All candidate housekeeping mRNAs presented M values below 1.5, the GeNorm set threshold, findings that are consistent with stability^22^. The Bestkeeper software points out inconsistency when SD is higher than 1.0, a pattern detected only for *Hprt* and *Pgam1* in the SC sample groups (Supplementary Table S2). NormFinder evaluates expression stability by analyzing the intra- and intergroup transcriptional variation of the candidate reference genes. An SD value lower than 0.5 is recommended to consider a gene a suitable housekeeping. *Ppia, Hprt*, *Pgam1* and *B2m* had SD values below 0.5, while the *Actb*, *Actg1* and *Gapdh* values lied above 0.5 in all sample groups. Moreover, *Pgam1* and *B2m* displayed SD higher than 0.5 in the SC group, *B2m* in CO and SC + CO, and *Gapdh* in CY, NC, HT, WT, SC e SC + CO groups. These results suggest that these candidate genes should not be considered suitable housekeepings in the mentioned groups and settings.

Based on the different used algorithms and considering all ranks yielded by the referred analyses, *Ppia* appeared as the most stably expressed housekeeping gene for analyses including all groups, as well as for the CY, SC, CY + NC and SC + CO groups. The data that supported this conclusion were yielded by NormFinder (SD = 0.22), GeNorm (M value = 0.53), RefFinder (Geomean = 1.00), ΔCt method (Mean SD = 0.80), Bestkeeper (CV = 3.24 and SD = 0.54) and DataAssist (Score = 0.68) (Supplementary Table S2). *Pgam1*, on the other hand, was the most stably expressed housekeeping gene for analyses involving the WT + CO groups, *Actb* for the NC group, and *Hprt* for analyses including the HT + WT groups as well as only HT animals (Table 1).

**Table 1:** Best housekeeping gene for each group of samples, each model and respective control, and all groups yielded by software analyses.

Groups	Best housekeeping genes identified by software analyses						Best housekeeping gene
	NormFinder	GeNorm	RefFinder	ΔCt method	Bestkeeper	DataAssist	
All	Ppia*	*Actb* + *Actg1*	*Ppia*	*Ppia*	*Hprt*	*Ppia*	*Ppia*
HT	*Pgam1*	*Hprt* + *Ppia*	*Pgam1*	*Pgam1*	*Hprt*	*Hprt*	*Hprt*/***Pgam1***
WT	*Ppia*	*B2m* + *Pgam1*	*Pgam1*	*Pgam1*	*Hprt*	*Ppia*	***Pgam1***
CY	*Ppia*	*Actg1* + *Ppia*	*Ppia*	*Ppia*	*Actb*	*Ppia*	***Ppia***
NC	*Pgam1*	*Actb* + *Pgam1*	*Hprt*	*Hprt*	*Actb*	*Ppia*	*Actb*/*Hprt*/***Pgam1***
SC	*Ppia*	*Actg1* + *Hprt*	*Ppia*	*Ppia*	*Gapdh*	*Ppia*	***Ppia***
CO	*Pgam1*	*Actg1* + *Ppia*	*Pgam1*	*Pgam1*	*Hprt*	*Actg1*	***Pgam1***
HT + WT	Pgam1*	*Hprt* + *Ppia*	*Actg1*	*Actg1*	*Hprt*	*Hprt*	*Hprt*
CY + NC	Pgam1*	*Actg1* + *Ppia*	*Ppia*	*Ppia*	*Actb*	*Ppia*	*Ppia*
SC + CO	Ppia*	*Actg1* + *Hprt*	*Ppia*	*Ppia*	*Actb*	*Ppia*	*Ppia*

*Best reference genes considering intra- and intergroup variations.

All, all samples; HT, haploinsufficient; WT, wild type; CY, cystic; NC, noncystic; SC, severely cystic phenotype; CO, control for the severely cystic phenotype. NormFinder (version 0.953; https://moma.dk/normfinder-software), GeNorm (version 2.2; https://genorm.cmgg.be/), BestKeeper (version 1.0; https://www.gene-quantification.de/bestkeeper.html), DataAssist (version 3.01; https://www.thermofisher.com/br/en/home/technical-resources/software-downloads/dataassist-software.html), the comparative ΔCt method and RefFinder (https://www.heartcure.com.au/reffinder/).

Genes in bold: higher frequency of appearance in softwares.

Taken together, our data ranked *Ppia* and *Hprt* as the most stable candidate housekeeping genes. *Gapdh*, on the other hand, was deemed as the least stable one, being associated with the following values: NormFinder (SD = 0.83), GeNorm (M value = 085), RefFinder (Geomean = 7.00), ΔCt method (Mean SD = 1.38), Bestkeeper (CV = 4.32 and SD = 0.82), and DataAssist (Score = 0.95) (Supplementary Table S2).

Since the selection of the most suitable housekeeping gene depends on the *Pkd1* deficiency model, it is important to conclude that *Ppia* was identified as the best housekeeping gene for the CY-NC and SC-CO pairs of samples while *Hprt* was the most suitable for HT-WT. Therefore, and interestingly, *Ppia* seems to be the best housekeeping in analysis involving models with cystic phenotypes (CY and SC), being associated with NormFinder (SD = 0.09), GeNorm (M value = 0.18), RefFinder (Geomean = 1.41), ΔCt method (Mean SD = 0.63), Bestkeeper (CV = 3.25 and SD = 0.84), and DataAssist (Score = 0.50) (Supplementary Table S2). *Pgam1*, in turn, appears as the most appropriate housekeeping gene when comparing noncystic controls (WT, NC and CO) (Table 1).

### Analysis of the best combination of housekeeping genes

The use of combined housekeeping genes is a strategy often employed to improve comparisons of relative target gene expression among groups. A comprehensive analysis of potential combinations of evaluated candidates, therefore, should be performed to establish the best anchors to quantify and compare gene expression. Ultimately, this approach constitutes a way of minimizing possible outliers and inappropriate differences between measurements.

The software packages recommend at least two genes to be used together as housekeeping genes. The best combination of candidate housekeepings for each of our group of samples, based on analyses yielded by different software packages, is shown in Table 2. Such analyses revealed that the *Actb* + *Hprt* and *Hprt* + *Ppia* pairs are the combinations most often identified as the best housekeeping gene options for comparisons including all groups, with the same statistical weight; *Actg1* + *Ppia* was the most frequent pair for the CY group; *Actb* + *Pgam1* for NC and WT; *Hprt* + *Pgam1* for HT; *Actb* + *Ppia* or *Actg1* + *Ppia* for SC; and *Actg1* + *Pgam1* for the CO group.

**Table 2:** Best combination of housekeeping genes for each group of samples, each model and respective control, and all groups yielded by software analyses.

Groups	Best pair of housekeeping genes identified by software analyses						Best pair of housekeeping genes	Best trio of housekeeping genes
	NormFinder	GeNorm	RefFinder	ΔCt method	Bestkeeper	DataAssist		
All	*Actb* + Pgam1*	*Actb* + *Actg1*	*Hprt* + *Ppia*	*Hprt* + *Ppia*	*Actb* + *Hprt*	*Hprt* + *Ppia*	*Actb* + *Hprt*/*Hprt* + *Ppia*	*Actb* + *Hprt* + *Ppia*
HT	*Hprt* + *Pgam1*	*Hprt* + *Ppia*	*Actg1* + *Pgam1*	*Actg1* + *Pgam1*	*Hprt* + *Ppia*	*B2m* + *Hprt*	*Hprt* + ***Pgam1***	*Actg1* + *Hprt* + ***Pgam1***/*Hprt* + ***Pgam1*** + *Ppia*
WT	*Pgam1* + *Ppia*	*B2m* + *Pgam1*	*Actb* + *Pgam1*	*Actb* + *Pgam1*	*Actb* + *Hprt*	*Pgam1* + *Ppia*	*Actb* + ***Pgam1***	*Actb* + ***Pgam1*** + *Ppia*
CY	*Pgam1* + *Ppia*	*Actg1* + *Ppia*	*Actg1* + *Ppia*	*Actg1* + *Ppia*	*Actb* + *B2m*	*Pgam1* + *Ppia*	*Actg1* + ***Ppia***	*Actb* + *Actg1* + ***Ppia***/*Actg1* + *Pgam1* + *Ppia*/*Actg1* + *B2m* + ***Ppia***
NC	*Pgam1* + *Ppia*	*Actb* + *Pgam1*	*Actb* + *Hprt*	*Actb* + *Hprt*	*Actb* + *Actg1*	*Pgam1* + *Ppia*	*Actb* + ***Pgam1***	*Actb* + *Hprt* + ***Pgam1***/*Actb* + *Pgam1* + *Ppia*
SC	*Actg1* + *Ppia*	*Actg1* + *Hprt*	*Actb* + *Ppia*	*Actb* + *Ppia*	*Actb* + *Gapdh*	*Actg1* + *Ppia*	*Actb* + ***Ppia***/*Actg1* + ***Ppia***	*Actb* + *Hprt* + ***Ppia***/*Actg1* + *Hprt* + ***Ppia***
CO	*Actg1* + *Pgam1*	*Actg1* + *Ppia*	*Gapdh* + *Pgam1*	*Actg1* + *Pgam1*	*Gapdh* + *Hprt*	*Actg1* + *Pgam1*	*Actg1* + *Pgam1*	*Actg1* + *Gapdh* + ***Pgam1***
HT + WT	*Hprt* + Pgam1*	*Hprt* + *Ppia*	*Actg1* + *B2m*	*Actg1* + *B2m*	*Actb* + *Hprt*	*B2m* + *Hprt*	*B2m* + *Hprt*	*Actg1* + *B2m* + *Hprt*
CY + NC	*Pgam1* + Ppia*	*Actg1* + *Ppia*	*Actg1* + *Ppia*	*Pgam1* + *Ppia*	*Actb* + *Hprt*	*Pgam1* + *Ppia*	*Pgam1* + *Ppia*	*Actg1* + *Pgam1* + *Ppia*
SC + CO	*Actg1* + Ppia*	*Actg1* + *Hprt*	*Actb* + *Ppia*	*Actg1* + *Ppia*	*Actb* + *Hprt*	*Actg1* + *Ppia*	*Actg1* + *Ppia*	*Actb* + *Actg1* + *Ppia*/*Actg1* + *Hprt* + *Ppia*

*Best reference genes considering intra- and intergroup variations.

All, all samples; HT, haploinsufficient; WT, wild type; CY, cystic; NC, noncystic; SC, severely cystic phenotype; CO, control for the severely cystic phenotype. NormFinder (version 0.953; https://moma.dk/normfinder-software), GeNorm (version 2.2; https://genorm.cmgg.be/), BestKeeper (version 1.0; https://www.gene-quantification.de/bestkeeper.html), DataAssist (version 3.01; https://www.thermofisher.com/br/en/home/technical-resources/software-downloads/dataassist-software.html), the comparative ΔCt method and RefFinder (https://www.heartcure.com.au/reffinder/).

Genes in bold: higher frequency of appearance in softwares.

Comparisons of the different *Pkd1*-deficient kidney tissues with their respective controls identified *Pgam1* + *Ppia* as the best pair of housekeeping genes for CY and NC, *B2m* + *Hprt* for HT and WT, and *Actg1* + *Ppia* for SC and CO. Additionally, *Actb* + *Hprt* + *Ppia* was the most frequent best trio identified for analyses including all groups; *Actg1* + *Pgam1* + *Ppia* for CY and NC, *Actg1* + *B2m* + *Hprt* for HT and WT, and *Actb* + *Actg1* + *Ppia* or *Actg1* + *Hprt* + *Ppia* for SC and CO (Table 2).

Notably, the identified best pair and trio combinations of housekeeping genes provided further support to the association of *Ppia* and *Pgam1* with the cystic and non-cystic phenotypes, respectively. Moreover, considering all samples together *Ppia* remains the most stable candidate housekeeping gene.

### Determination of the optimal number of housekeeping genes

The use of an optimal number of reference genes is important to save samples and primers, select and validate housekeeping genes, classify samples, group genes and monitor time-dependent processes. While the selection and use of stable housekeeping genes is often limited to only one, in most of such cases the stability of gene expression analysis could improve with the inclusion of one or more additional housekeeping controls. In this scenario, in the current study we established the minimum number of housekeeping genes that should be used in gene expression analysis involving each of our groups, each model with its respective control, and all groups.

**Figure 3:**
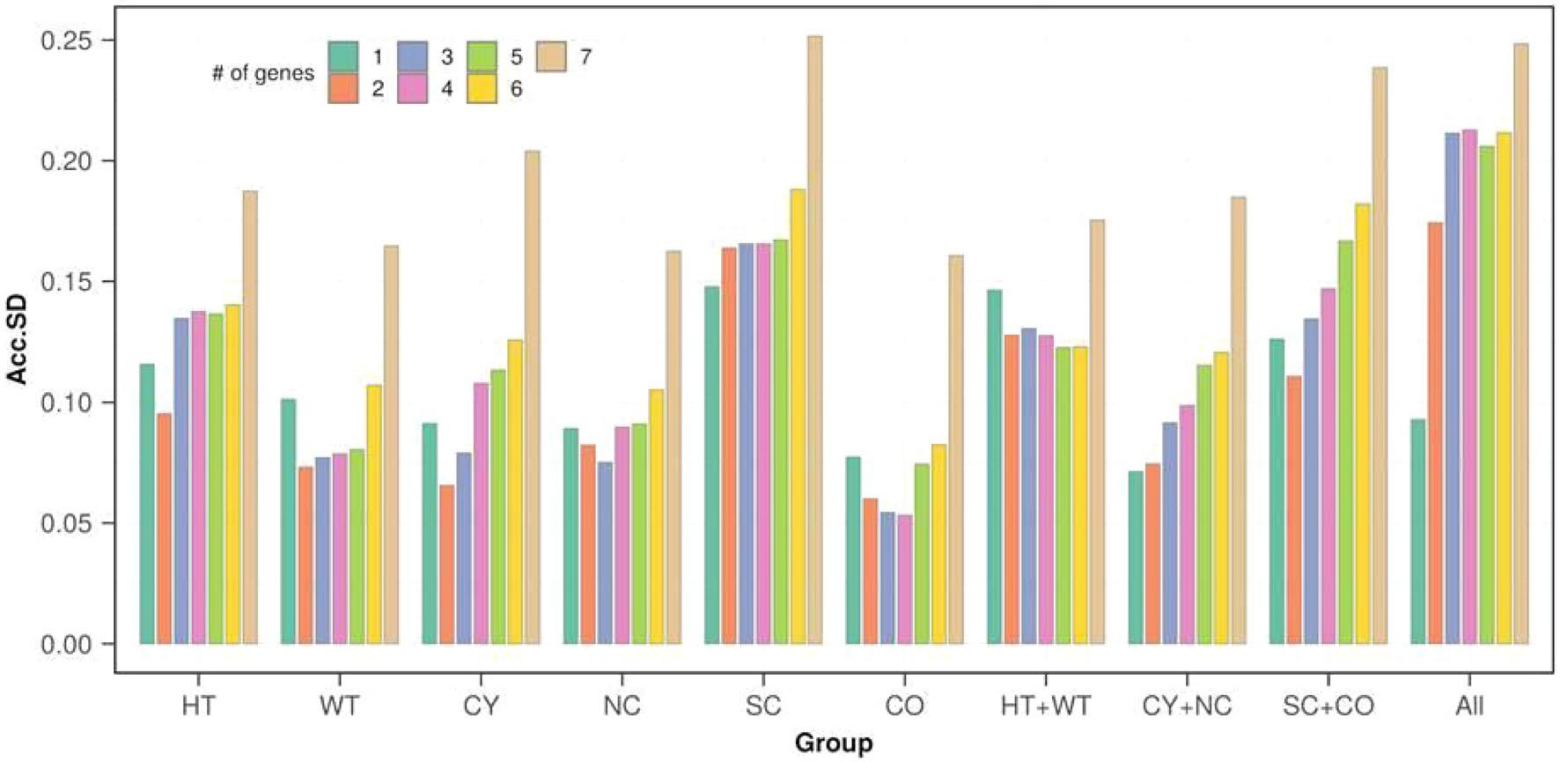
Optimal number of reference genes for each dataset according to GenEx analyses. Accumulated standard deviation (Acc.SD) for the five candidate housekeeping genes in all groups of samples allows estimating the ideal number of genes for normalization. Acc.SD was calculated with the GenEx software package (version 6; http://www.multid.se). Lower values of Acc.SD indicate the optimal number of reference genes. HT, *Pkd1*-haploinsufficient; WT, wild type; CY, cystic; NC, noncystic; SC, severely cystic; CO, control for the severely cystic phenotype.

**Figure 4:**
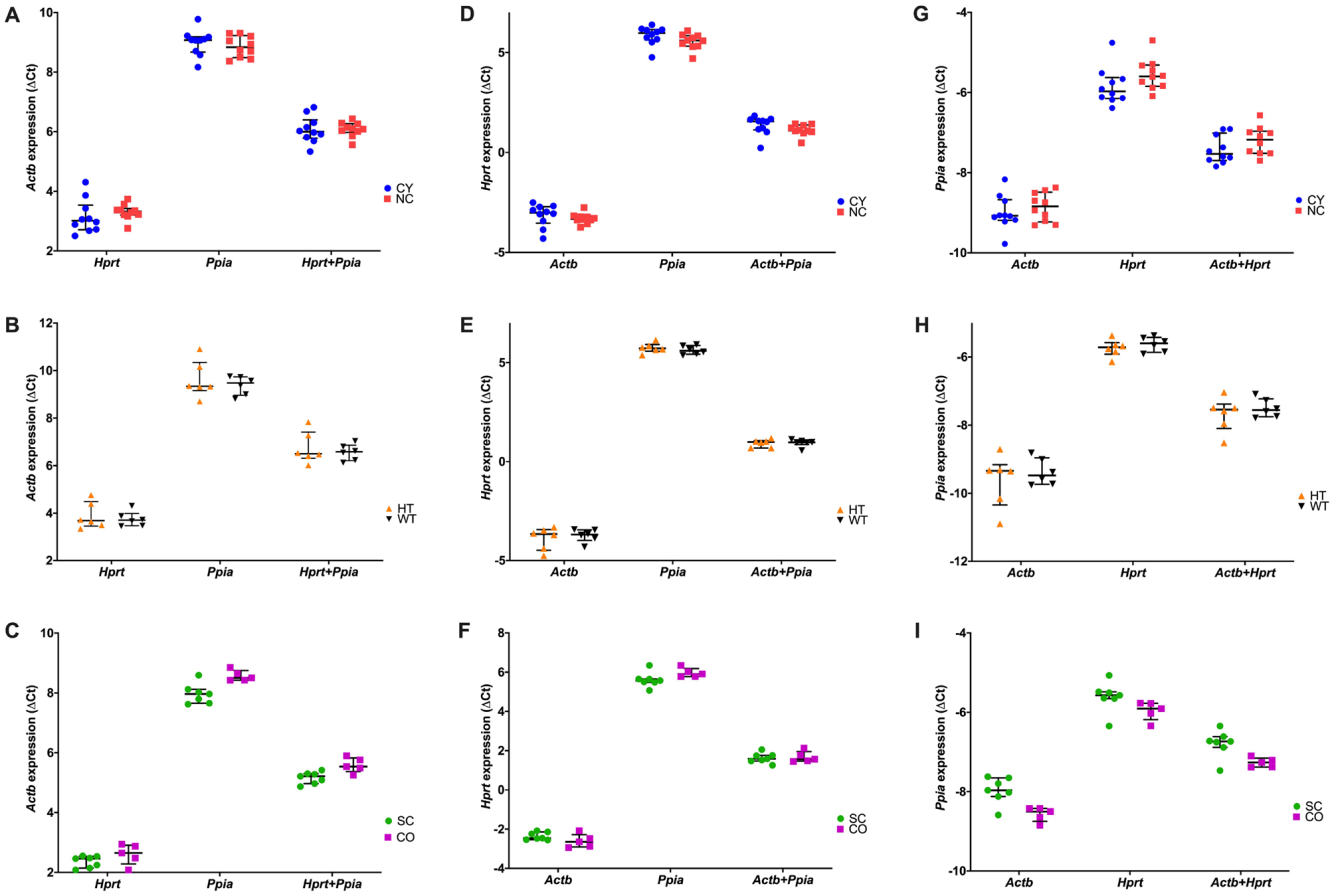
The ΔCt values of the three best candidate housekeeping genes: *Actb* (**A–C**), *Hprt* (**D–F**) and *Ppia* (**G–I**). A lower threshold value (Ct) indicates a higher gene expression level. The median values are expressed as horizontal lines while the error bars represent interquartile ranges. CY, cystic; NC, noncystic; HT, haploinsufficient; WT, wild type; SC, severely cystic phenotype; CO, control of severely cystic phenotype. *Actb*, target expression normalized by *Actb*; *Hprt*, target expression normalized by *Hprt*; *Ppia*, target expression normalized by *Ppia*; *Actb* + *Hprt*, target expression normalized by *Actb* + *Hprt*; *Actb* + *Ppia*, target expression normalized by *Actb* + *Ppia*; *Hprt* + *Ppia*, target expression normalized by *Hprt* + *Ppia*. Comparisons between the *Pkd1*-deficient model and its respective control were performed with the Mann–Whitney U test. P < 0.05 was not detected for any of the comparisons.

The GenEx software package was used to calculate the Acc.SD for the seven candidate housekeeping genes and to determine the optimal number of reference genes to be used for each dataset (Fig. 3). Based on the 0.15 Acc.SD cut-off, *Ppia* was identified as the most stable candidate gene for normalization when considering all sample groups (All; Fig. 4). The addition of another gene led to a cut-off greater than 0.15, demonstrating that the insertion of a second normalizer in the gene expression analysis would increase the Acc.SD. Moreover, the addition of more than one gene led to a cut-off greater than 0.2, indicating that the insertion of a third gene would increase even more the Acc.SD. Applying a global analysis and based on the lowest Acc.SD value, the best normalizers for each sample group were found to be: two (*Actg1* + *Ppia*) was the optimal number of housekeeping genes to be considered for the CY group, three (*Actb* + *Hprt* + *Pgam1*) for the NC group, two (*Hprt* + *Pgam1*) for HT, two (*Actb* + *Pgam1*) for the WT group, one (*Ppia*) for SC, and three (*Actg1* + *Gapdh* + *Pgam1*) for the CO group. Importantly, we observed that different numbers of housekeeping genes should be used when comparing the renal tissue of each *Pkd1*-deficient model with its respective control: one (*Ppia*) for CY + NC, two (*B2m* + *Hprt*) for HT + WT, and two (*Actg1* + *Ppia*) for SC + CO.

### Intergene expression normalization among the top three candidate housekeeping genes

An optimal housekeeping gene should present expression stability, displaying low expression variability with respect to other housekeeping genes. This property can be evaluated by normalizing their expression by each other’s. To evaluate this aspect for the best candidate housekeeping genes, we used as the third strategy different algorithms to normalize the expression levels (Ct) of the top three candidates (*Actb* + *Hprt* + *Ppia*) to each other’s expression (Fig. 4). Their expression levels did not differ between any two-sample groups. These results suggest that any of these three genes are suitable to be employed as housekeeping genes among the analyzed samples. Interestingly, the presence of *Ppia* as a housekeeping gene decreased the gene expression dispersion of both *Actb* and *Hprt* (Fig. 4).

### Correlation of mRNAs expression between pairs of the top three candidate housekeeping genes

**Figure 5:**
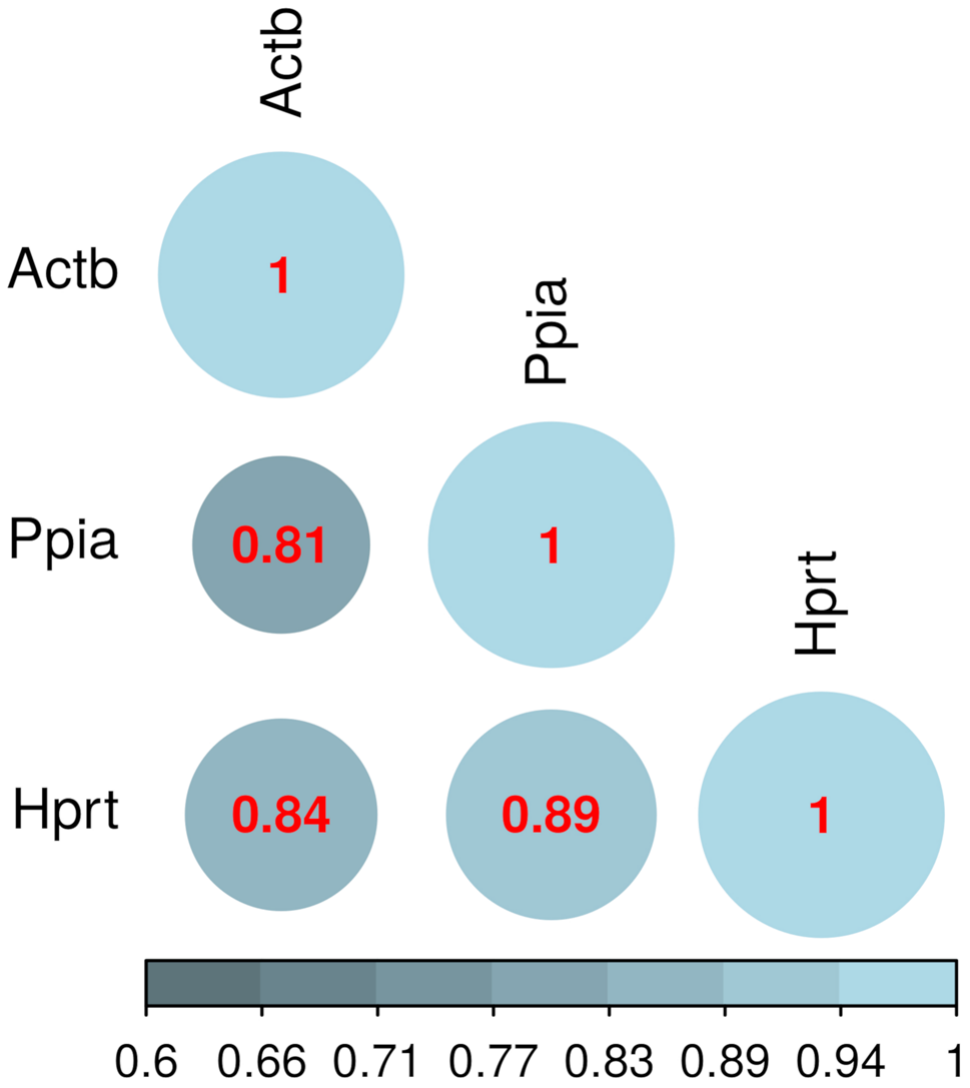
Pair correlation matrix between the number of amplification cycles of the best housekeeping candidate genes *Actb, Hprt* and *Ppia*. Circle color and radius are proportional to the Spearman’s rank correlation coefficient (significance level: p ≤ 0.05).

Pair correlation analysis between the best housekeeping genes can provide options to choose relevant housekeeping genes for gene expression studies. This approach was applied to our models orthologous to ADPKD associated with different profiles of *Pkd1* deficiency, displaying or not a cystic phenotype. Such correlation analyses were performed using the mRNAs expression data yielded by all evaluated kidney samples. The expression levels (Ct) of the three best candidate housekeeping genes revealed a very strong correlation between *Hprt* and *Ppia* (*ρ* = 0.89, p < 0.05, Fig. 5) and a strong correlation between *Actb* and *Hprt* (*ρ* = 0.84, p < 0.05, Fig. 5). In addition, a moderate correlation was observed between *Actb* and *Ppia* (*ρ* = 0.81, p < 0.05, Fig. 5). These results showed that the *Actb*, *Hprt* and *Ppia* expression profiles are correlated in all the samples herein evaluated and can be used together as suitable housekeeping genes.

### Validation of the best candidate housekeeping genes by normalizing expression of *Stat3* target gene

**Figure 6:**
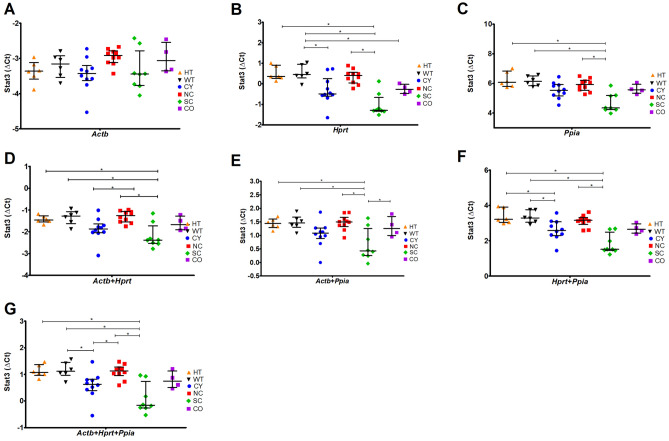
The ΔCt values of *Stat3* expression target gene normalized by different combinations of the three best candidate housekeeping genes (*Actb*, *Hprt and Ppia*) (**A**) *Stat3/Actb*, target expression normalized by *Actb*; (**B**) *Stat3/Hprt*, target expression normalized by *Hprt*; (**C**) *Stat3/Ppia*, target expression normalized by *Ppia*; (**D**) *Stat3/Actb* + *Hprt*, target expression normalized by *Actb* + *Hprt*; (**E**) *Stat3/Actb* + *Ppia*, target expression normalized by *Actb* + *Ppia*; (**F**) *Stat3/Hprt* + *Ppia*, target expression normalized by *Hprt* + *Ppia*, (**G**) *Stat3/Actb* + *Hprt* + *Ppia* target expression normalized by *Actb* + *Hprt* + *Ppia*. The median values are represented as horizontal lines and the error bars represent interquartile ranges. CY, cystic; NC, noncystic; HT, haploinsufficient; WT, wild type; SC, severely cystic phenotype; CO, control for the severely cystic phenotype. *p < 0.05 by Kruskal–Wallis with Dunn’s post-hoc test, followed by FDR correction.

In order to validate the expression stability of the three best candidate housekeeping genes, the relative expression of the *Stat3* target gene was assessed using different combinations of *Actb*, *Hprt* and *Ppia* normalizers (Fig. 6). *Stat3* was selected for this purpose because it is a well-defined gene with increased expression in ADPKD and in murine polycystic kidney disease models.

The *Stat3* expression level was consistent with upregulation in SC kidneys compared to its control CO when normalized by *Ppia* expression (Fig. 6C). Trends of upregulation were also observed in SC kidneys compared to CO when normalized by other combinations of the best candidate housekeeping genes (Figs. 6B, 6D, 6E, 6F and 6G). Trends of *Stat3* upregulation in CY compared to their control kidneys were also observed when its expression was normalized by different combinations of *Actb*, *Hprt* and *Ppia* (Figs. 6A, 6B, 6D, 6E, 6F and 6G). In contrast, *Stat3* expression was not statistically different between HT and WT kidneys regardless the housekeeping gene combination used for normalization (Fig. 6). Our results also suggest that the SC kidneys, and likely their controls CO, have expression profiles slightly different from the other analyzed mouse model kidneys. Taken together, the use of *Ppia* showed to be the most suitable housekeeping gene among all considered sample groups.

Many studies with other animal models, including kidney tissues, have used *Gapdh* as the housekeeping gene, assuming unaltered expression^20,23–25^. Given the weakness of *Gapdh* as a housekeeping gene observed in our models, it is essential that this gene be analyzed and compared to other potential housekeeping gene normalizers. Indeed, when we analyzed *Stat3* expression using *Gapdh* as the housekeeping normalizer, we did not find different expression levels between CY and NC, HT and WT, and SC and CO (Supplementary Figure S2). These finding are in disagreement with the trends of *Stat3* upregulation detected in CY kidneys compared to NC and in SC compared to CO evidenced when our housekeeping gene combinations were applied.

### Expression of the best candidate housekeeping genes does not correlate with the kidney weight/body weight ratio in CY mice

Since analyses involving mouse models orthologous to human ADPKD often require assessment of severity of the cystic phenotype or cyst growth, we reasoned that the identified housekeeping genes should have their stability evaluated with respect to the renal cyst burden, reflected in kidney size/weight. This approach could allow the investigation whether the disease stage, progression and severity significantly modify the expression patterns of potential housekeeping genes. To accomplish this task, we sought for potential correlations between the expression levels of *Actb*, *Hprt* and *Ppia* and kidney weight/body weight ratio (KW/BW) in the CY group. The expression levels of *Actb*, *Hprt* and *Ppia*, however, did not correlate with KW/BW (*ρ* = 0.05, p < 0.871, Supplementary Figure S3A; *ρ* = −0.22, p < 0.42, Supplementary Figure S3B; and *ρ* = 0.44, p = 0.09, Supplementary Figure S3C, respectively). This lack of correlations strongly suggests that the cystic burden does not significantly influence the expression level of the analyzed housekeeping genes, further supporting their use as controls in studies involving animals with different severities of renal cystic phenotypes.

## Discussion

*PKD1*/*PKD2* pathogenic mutations deregulate a number of downstream pathways that aberrantly affect major cellular properties and contribute to the ADPKD phenotype. Such pathways encompass mTOR, cMYC, STATs, cAMP and intracellular calcium homeostasis, promoting cyst epithelial cell proliferation, apoptosis, epithelial secretion, and cellular metabolic rewiring^26^ which in turn constitute logical targets for the development of therapeutic interventions. Given there is no widely accepted single model of ADPKD for therapeutic testing at present, most agents are tested in multiple preclinical models^27^. Since orthologous animal models with slowly progressive renal cystic disease reproduce more closely the human disease, they are more appropriate to unravel mechanisms underlying ADPKD pathogenesis and generate useful biomarkers^28^.

To systematically evaluate the complexity of target gene expression analyses in in vivo models orthologous to ADPKD and propose the best standards for such studies, we aimed to establish the most appropriate housekeeping genes to be employed in three mouse models with distinct profiles of *Pkd1* gene deficiency, in different experimental scenarios. While HT mice show almost exclusively *Pkd1*^+/−^ renal cells but do not display cysts, reproducing the background cell environment found in ADPKD type 1 patients, CY mice have renal cysts presumably formed by *Pkd1*^*−/−*^ cells, reproducing the ADPKD type 1 cystic phenotype and its expected consequences. Notably, CY mice have preserved GFR at the evaluated ages (12 weeks). To complete the diverse phenotypes associated with *Pkd1* deficiency, we included in our systematic assessment, an early, severely cystic animal model, the *Pkd1*^V/V^ mouse (SC).

One reason justified the use of the SC (15 days) mouse as the model of highly cystic burden and another reason supported/validated its use as such a model. The first reason is because SC mice have a very short survival due to their fast progression to renal failure^29^. We had to choose an age, therefore, at which the significant majority of these animals would be still alive; so the chosen age was 15 days. In contrast, CY mice had to be analyzed at a significantly higher age, to have enough time to present an adequate cystic phenotype. In this context, we chose to work with 10–12-week-old CY and NC mice, as well as 10–12-week-old HT and WT animals; CY animals display a mild to moderate renal cystic phenotype which has been shown to be associated with preserved renal function even at ages of a few more weeks than the age range used in the current study^30,31^. The other reason, which supported/validated our decision, was the finding that the housekeeping gene expression profiles detected in CO mice (control wild-type animals with 15 days of life) did not significantly differ from the other controls WT and NC (*PPIA* with median Cts lying between 16 and 17, *HPRT* between 21–24 and *ACTB* between 24–27). Moreover, *Stat3* expression did not significantly differ among CO, WT and NC kidneys in all but one normalization. Finally, we assessed the correlation between the best candidate housekeeping genes and KW/BW ratio in the CY group of samples. The detected absence of correlation between the expression levels of the best ones and the KW/BW ratio in the CY group suggests that the level of cystic involvement does not lead to significant changes in housekeeping gene expression.

While the use of non-validated endogenous control genes in gene expression studies results in unreliable data, a universal, invariably expressed gene is unlikely to exist^32^. This hypothetical control gene, in fact, may not even exist within individual tissues and cell types. In this context, a more adequate purpose is to identify the most reliable gene or set of genes to be applied in each experimental setting. Although several studies have evaluated gene expression profiles to identify new biomarkers and therapeutic targets for ADPKD^26,33^, to the best of our knowledge, no study to date has addressed the appropriateness of housekeeping gene usage in animal models orthologous to ADPKD. The present study identified *Ppia* as the best housekeeping gene for CY + NC and SC + CO groups, while *Hprt* was the best for the HT + WT group.

Cui et al. (2009)^34^ have previously analyzed endogenous genes as potentially useful housekeeping genes for analyses of target gene expression in kidney tissue samples of *cpk* mice, a well-characterized recessive cystic kidney disease model^34^. They studied the expression of 16 commonly used housekeeping genes in seven mildly and seven severely affected whole kidney tissue samples using TaqMan RT-qPCR assays and Affymetrix GeneChip arrays, normalized and tested for overall variance and equivalence of the means. Both statistical approaches and both TaqMan- and GeneChip-based methods converged on 3 out of the 4 top-ranked genes (*Ppia*, *Gapdh* and *Pgk1*) that displayed the most constant expression levels across the assessed phenotypes. Such results led to the conclusion that a combination of the top-ranked genes would provide suitable endogenous internal control for gene expression studies in *cpk* kidney tissues across a wide range of disease severity. The different disease etiology, however, precludes the application of these data to gene expression studies performed in in vivo models orthologous to ADPKD. The rationale of the current study was to run the first assessment of candidate housekeeping genes for normalization of mRNA expression by RT-qPCR in kidney tissue of mouse models orthologous to ADPKD with distinct patterns of *Pkd1* deficiency.

Leal et al. (2016)^35^ has successfully evaluated the suitability of reference genes in meniscus samples in pathological and control conditions using the software packages applied in our current analysis^35^. These investigators revealed *HPRT1* as the best single reference gene but as expected, showed that two or more reference genes should be used for gene expression normalization instead. According to the samples tested and proposed comparisons, the appropriate housekeeping gene combination included *HPRT1* + *TBP*, *HPRT1* + *GAPDH*, or *HPRT1* + *TBP* + *GAPDH*. Other authors suggested that *PPIA* and *RPS13*, especially in combination, were the best suitable references to normalize gene expression in ccRCC tissues as compared to classical reference genes such as *beta-Actin, GAPDH*, *18S* or *B2M*^36^. Therefore, such reports served as anchors for our present investigation about the stability of seven candidate genes selected from previous studies (*Actb*, *Actg1*, *B2m*, *Gapdh*, *Hprt*, *Pgam1* and *Ppia*)^24,34,37–41^ in kidneys samples of *Pkd1*-deficient noncystic and cystic mouse models. The stability of gene expression was analyzed using distinct statistical models, including a pairwise comparison model, geNorm, and an ANOVA-based model, NormFinder.

Each of the applied algorithms ranked the best candidate reference genes, identifying *Ppia* as the most stable and reliable housekeeping gene, while *Gapdh* was least stable for all kidney samples. *Gapdh* is the most commonly used gene as endogenous control in RT-qPCR analyses, since its expression is usually constant^23–25,41,42^. Using a similar approach, a recent report evaluated six commonly used reference genes (*Actb, B2m, Gapdh, Hmbs, Hprt and Ppia*) to identify the most constantly expressed gene under the influence of testosterone in rat^43^. This study showed that *Hmbs* and *Ppia* were the most stably expressed genes in the hypothalamus while *Hmbs* and *Gapdh* appeared to be the most stable genes in kidneys, indicating that in this setting the *Gapdh* expression profile was more stable than in our case. Concentrations of *GAPDH*, however, may vary among individuals^44^, during pregnancy^45^, according to developmental stage^46,47^ and during the cell cycle^48^. Other reports also documented such a limitation^49–53^. Therefore, the recognition that the expression of *Gapdh* may exhibit tissue-specific regulationis consistent with our results and emphasizes the need for validation of commercially available control assays.

In line with the aforementioned raised concerns, our findings revealed that the selection of appropriate housekeeping genes and consequent use of *Ppia* as the reference, allowed the detection of differences between groups regarding *Stat3* expression, thought to be upregulated in kidney cysts and associated with PKD progression in mouse models^8,54–60^. On the other hand, the normalization using *Gapdh* showed no difference. Interestingly, despite the different ages between the CY and SC groups, normalization using *Ppia* showed very close expression profiles in these two groups, decreasing the expression dispersion observed with *Actb* and *Hprt*. These data suggest that *Ppia* is a good expression normalizer for models associated with renal cystic phenotypes, a piece of information that may be highly relevant to investigate and compare different cystic disease stages, progression and severity. On the other hand, *Pgam1* was associated with the *Pkd1*-haploinsufficient non-cystic model, suggesting that this might be a more appropriate normalizer to be used in studies involving *Pkd1*-deficient non-cystic renal phenotypes.

One limitation of the present study relies on the selection of only seven candidate housekeeping genes to be tested for validation, a process that was based on data available in previous studies. We believe, therefore, that further research is still required is this field, since the evaluation of additional candidate housekeeping genes in additional *Pkd1*-orthologous mouse models and experimental conditions are likely to improve the specificies of gene normalization in this biological scenario. Moreover, the pipeline adopted in the present study should be further tested using other models as well.

In conclusion, our findings established *Ppia* as the most appropriate housekeeping gene for comparisons involving a cystic model and its respective control, *Hprt* for non-cystic *Pkd1*-haploinsufficient and wild-type mice, and *Ppia* for a severely cystic model and its corresponding wild-type control. Overall, our analysis identified *Ppia* as the best and the most stable housekeeping gene in the comprehensive *Pkd1*-deficiency scenario, while *Gapdh* was the least stable in the three evaluated mouse models. Such data will allow more robust and reliable analyses of target gene expression in kidney tissue of *Pkd1*-deficient mouse models, a reality that will contribute to the elucidation of the role of different genes in different scenarios related to ADPKD or *Pkd1* biology. Normalizing to a suitable housekeeping gene or sets of them can not only remove artifactual differences due to sampling and quality of mRNA but also identify real changes in gene expression levels.

## Methods

### Ethical statement

The authors confirm that all experiments were carried out in accordance with ARRIVE guidelines and regulations (https://arriveguidelines.org). "The ethical approval for all animal care and procedures were given by the Ethic Committee on Animal Use of the Federal University of Sao Paulo—Brazil (CEUA/UNIFESP), protocol number CEUA 4558140219."

### ADPKD mouse models

The three mouse models were maintained on the C57BL/6 strain background, an important requirement for the performance of our study. Two models were evaluated at 10–12 weeks of age: a renal cystic mouse (*Pkd1*^flox/flox^:*Nestin*^cre^/*Pkd1*^flox/−^:*Nestin*^cre^, CY, n = 10; and their corresponding noncystic controls *Pkd1*^flox^/^flox^/*Pkd1*^flox^/^-^, NC, n = 10) and a *Pkd1*-haploinsufficient noncystic mouse (*Pkd1*^+/−^, HT, n = 6) and its respective wild-type control (*Pkd1*^+/+^, WT, n = 6). The third model was assessed at 15 days of life due to its severely renal cystic phenotype (*Pkd1*^V/V^, SC, n = 7) along with its same-age wild-type control (CO, n = 5). It must be noted that the WT and CO controls harbor the same genotype (*Pkd1*^+/+^) but were analyzed at different ages. Only kidneys from male animals were analyzed in order to avoid potential gender-related experimental variability. The mice were genotyped using specific PCR reactions^21^.

The CY mice, homozygous for a *Pkd1*-floxed allele (*Pkd1*^flox^) or compound heterozygous *Pkd1*^flox/−^, display a mosaic pattern of full gene inactivation induced by a Nestin-Cre transgene through excision of exons 2–4 (*Pkd1*^flox/flox^:*Nestin*^cre^)^30,41,61–63^. The HT model is heterozygous for a *Pkd1* null allele, characterized by early transcriptional interruption^30,64^, and develops no renal cysts by 12 weeks of age. The SC model is homozygous for the *Pkd1* knockin T3041V allele, which prevents the autoproteolytic cleavage of PC1 at the GPS site^29,30^. *Pkd1*^V/V^ animals have no gross phenotype by postnatal day (P) 6 but develop rapid and progressive distal nephron cysts thereafter. This severe renal phenotype, which eventually leads to renal failure, is responsible for the early mortality that occurs between the 2nd and 6th week^29^.

The mice were fed ad libitum and housed at constant ambient temperature in a 12-h light cycle. Animal procedures were approved by the Internal Biosafety Commission of Genetically Modified Organisms of the University of São Paulo School of Medicine and by the Universidade Federal de São Paulo (UNIFESP) Research Ethics Committees. SC (*Pkd1*^V/V^) and its wild-type control (CO) animals were euthanized by cervical dislocation, whereas the other animal groups were euthanized with intraperitoneal thiopental (0.4 mg/g of body weight). Their kidneys were appropriately harvested for RT-qPCR analyses. All experiments were conducted in accordance with international standards of animal care and experimentation. Both kidneys were collected and stored at − 80 °C for further use^21^.

### Housekeeping genes

Using the terms “genes” and “polycystic and reverse transcriptase polymerase chain reaction” in the PubMed search, we found seven articles employing tissue samples from human ADPKD and animal ADPKD-orthologous kidneys. We also included housekeeping genes selected from other tissues in the literature^24,34,37–41^, namely *Actb, Actg1, B2m, Gapdh, Hprt, Pgam1* and *Ppia*. All of these seven genes are constitutively expressed in kidney tissue of mouse models orthologous to ADPKD, have independent cellular functions, and are assumed not to be co-regulated.

### Primers design

The oligonucleotide primers used to detect the gene-related products were designed based on the corresponding mRNA sequences (http://www.ncbi.nlm.nih.gov/nucleotide), using the Primer-Blast program (http://www.ncbi.nlm.nih.gov/tools/primer-blast/). This tool allows the design of primers taking into account alignments, even if partial, with other sequences potentially amplifiable by the primers, an evaluation that assesses all relevant sequences together. The Oligoanalyzer program (http://www.idtdna.com/analyzer/applications/oligoanalyzer) was also used to predict possible formation of stable secondary structures (hairpins) and primer dimers (homo and heterodimers, in the case of stable 3' structure), virtually guaranteeing the amplification of single products (no primer dimers and other products). To avoid amplification of genomic DNA due to potential contamination, the designed primers flanked exon–intron and intron–exon junctions expected to amplify products > 1000 bp from genomic DNA in all analyzed cases. To warrant high amplification efficiency, a maximum product size of 150 bp was established.

### RNA extraction

Renal tissue lysis was performed using zirconia beads (Interprise, USA) and the Precellys (BioAmerica, USA) homogenizer. TRIzol (Life Technologies, USA) was employed for total RNA extraction according to the manufacturer’s protocol. The RNA quantity and purity were determined using the NanoVue spectrophotometer (GE Healthcare Life Sciences, USA) and analyzed with the Agilent 2100 Bioanalyzer 6000 Nanochip (Agilent Technologies Inc., Waldbronn, BW, Germany). We treated 2 µg of total RNA with DNase (RQ1 RNase-free DNase; Promega, USA), to avoid genomic DNA contamination. Total RNA was stored at -80 °C until further use^21^.

### cDNA preparation and RT-qPCR

Complementary DNA (cDNA) was synthesized from 2 µg of total RNA using the High-Capacity cDNA Reverse Transcription Kit (Applied Biosystems), according to the manufacturer`s instructions.

Gene expression was performed in triplicate using SYBR Green (Applied Biosystems) in the QuantStudio7 qPCR system according to the manufacturer`s instructions. Primer sequences for the 7 candidate housekeeping genes and the target gene are shown in Supplementary Table S1. Gene expression quantification was performed in the same run for each sample to eliminate technical variation. Previously, we validated the amplification efficiencies of each primer, which were within the 90%-110% range.

### Analysis of housekeeping gene expression stability

The tissue samples were classified into 7 distinct groups: (1) CY, including the cystic kidney samples; (2) NC, non-cystic kidney samples; (3) HT, *Pkd1*-haploinsuficient kidney samples; (4) WT, wild-type kidney samples; (5) SC, severely cystic kidney samples; (6) CO, early-life, wild-type kidney samples; and (7) All, including all kidney samples. The three mouse models with distinct patterns of *Pkd1* deficiency were compared with their respective control tissues: CY vs NC, HT vs WT and SC vs CO.

RT-qPCR Cts were manually settled as 0.02 while the mean Ct values of the three technical replicates were imported to six algorithms: NormFinder (version 0.953; https://moma.dk/normfinder-software),65 GeNorm (version 2.2; https://genorm.cmgg.be/),22 BestKeeper (version 1.0; https://www.gene-quantification.de/bestkeeper.html),66 DataAssist (version 3.01; https://www.thermofisher.com/br/en/home/technical-resources/software-downloads/dataassist-software.html), the comparative ΔCt method^12^ and RefFinder (https://www.heartcure.com.au/reffinder/)67 following the authors’ recommendations. These software packages were used to determine the relative expression stability of the candidate housekeeping genes and to generate a ranking for the best ones.

The NormFinder software is a Microsoft Excel-based application that evaluates the expression stability of candidate reference genes. The stability value is calculated by analyzing their intra- and intergroup transcriptional variation. A lower variation in the expression levels indicates more stable gene expression (low stability value)^65^. The GeNorm program determines the gene expression stability value M according to the average pairwise variation between one particular gene and all other candidate genes. The most stable gene expression yields the lowest M value^22^. The BestKeeper program calculates the standard deviation (SD) and the coefficient of variance (CV) of the Ct levels, and applies the Pearson correlation coefficient to estimate the inter-gene relations of all possible candidate gene pairs^66^. The DataAssist software, in turn, determines a score for the best reference genes based on the GeNorm algorithm. It uses the RQ to calculate the stability value of each gene. A lower score indicates more stable expression (Thermo Fisher, USA). The comparative ΔCt method is based on a comparison of the relative expression of possible gene pairs within each sample. Gene stability is ranked according to the reproducibility of the gene expression differences in the analyzed samples^12^. Lastly, the RefFinder software integrates the NormFinder, GeNorm, BestKeeper, and the comparative ΔCt method assigning an appropriate weight to each gene, calculating the geometric mean of these weights, and generating a rank of the best candidate reference genes^67^. The optimal number of reference genes was selected using the GenEx software package (version 6; http://www.multid.se).

### Statistical analysis

The Shapiro–Wilk test showed that the Ct values of the candidate housekeeping genes were not normally distributed, so that the results are expressed as median and interquartile range (IQ). The ΔCt values were determined by: Ct(target gene)−Ct(housekeeping gene) or Ct(target gene)−mean[Ct(housekeeping gene 1);Ct(housekeeping gene 2)].

To evaluate potential expression differences of *Actb, Actg1, B2m, Gapdh, Hprt, Pgam1, Ppia and Stat3* among the sample groups, the Kruskal–Wallis with Dunn’s post-hoc test, followed by FDR correction using the Benjamini–Hochberg method, was used. Comparisons between the *Pkd1*-deficient model model and its respective control were performed with the Mann–Whitney U test.

Lastly, the Spearman correlation test was employed to verify potential correlations among the expression levels of *Actb*, *Hprt* and *Ppia*, and between the expression levels of such genes and kidney weight in CY cystic kidney samples. A value between 0.30–0.50 was determined as a weak correlation, 0.50–0.70 as moderate, 0.70–0.90 as strong, and 0.90–1.00 as a very strong correlation^68^.

## Supplementary Information

Supplementary Information.

## Data Availability

All data including supporting datasets are made available as main figures or supplementary information files.
