# Supplementary material for: Ppia is the most stable housekeeping gene for qRT-PCR normalization in kidneys of three Pkd1-deficient mouse models
Source: Sci Rep. 2021 Oct 5;11:19798. doi: 10.1038/s41598-021-99366-x (PMC8492864; doi:10.1038/s41598-021-99366-x)
Supplement: Supplementary file 1 — Supplementary Information. [file 41598_2021_99366_MOESM1_ESM.doc]

***Ppia* is the most stable housekeeping gene for qRT-PCR normalization in kidneys of three *Pkd1*-deficient mouse models**

Muñoz JJ^1^*, Anauate AC^1^*, Amaral AG^2^, Ferreira FM^3^, Watanabe EH^2^, Meca R^1^, Ormanji MS^1^, Boim MA^1^, Onuchic LF^2^*& Heilberg IP^1^*

^1^Nephrology Division, Department of Medicine, Universidade Federal de São Paulo, São Paulo, Brazil.

^2^Divisions of Molecular Medicine and Nephrology, University of São Paulo School of Medicine, São Paulo, Brazil.

^3^Division of Pathology, University of São Paulo School of Medicine, São Paulo, Brazil

*These authors contributed equally.

**Corresponding author:**

Ita Pfeferman Heilberg, M.D., PhD

Nephrology Division

Universidade Federal de São Paulo

Rua Botucatu 740 - Vila Clementino

04023-900

São Paulo - Brazil

Tel + 5511-55764848 ext 2465

E-mail address: ita.heilberg@gmail.com

**Running title:** *Ppia* housekeeping gene in *Pkd1* deficient mouse models.

**Table of Contents:**

**Supplementary Information**

1. **Supplementary Figure S1**
2. **Supplementary Figure S2**
3. **Supplementary Figure S3**
4. **Supplementary Table S1**
5. **Supplementary Table S2**
6. **Supplementary Table S3**
7. **Supplementary Table S4**


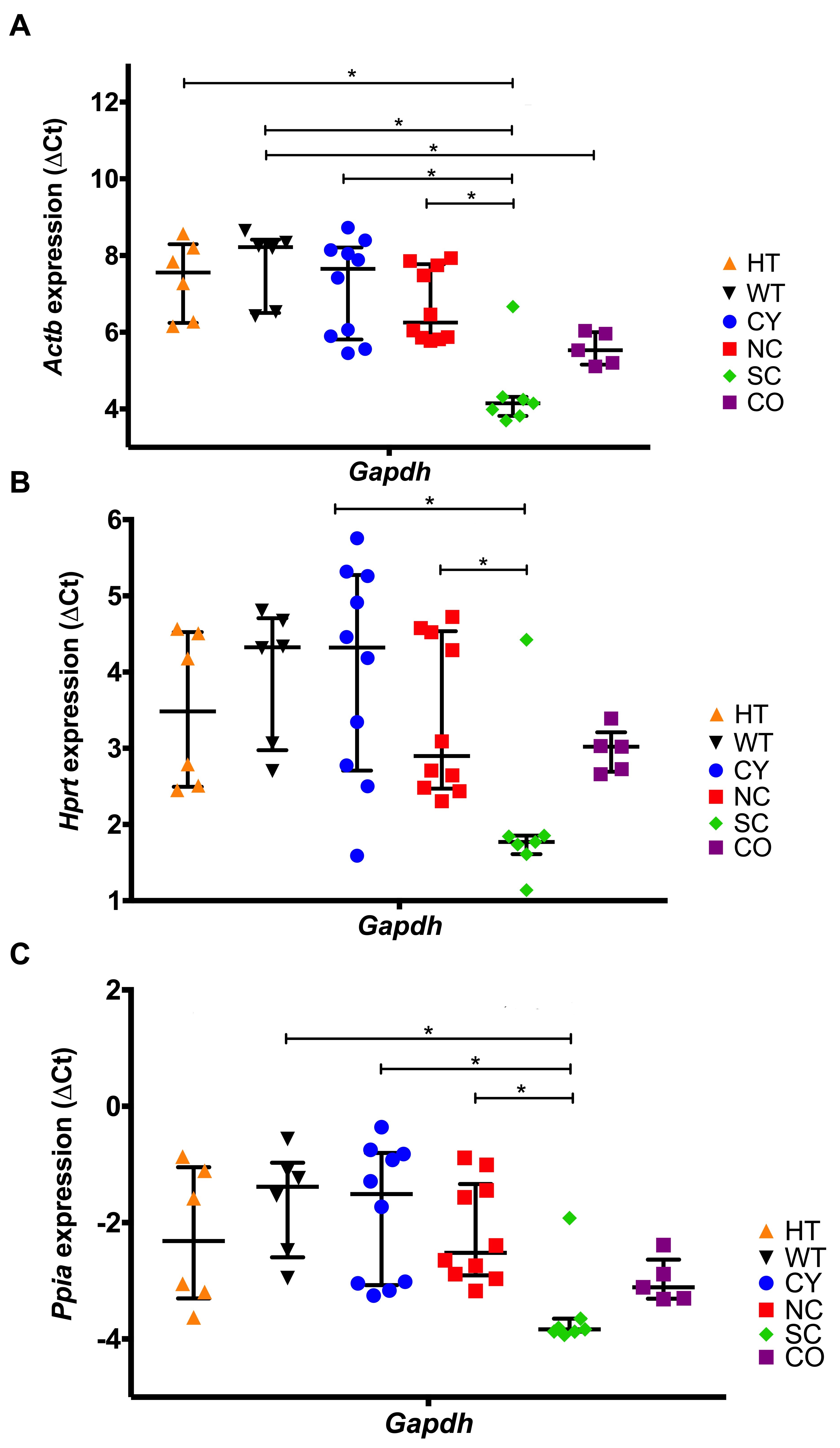


**Supplementary Figure S1.** The ΔCt values of *Actb* (**A**), *Hprt* (**B**) and *Ppia* (**C**) candidate housekeeping genes normalized by *Gapdh*. A lower threshold value (Ct) indicates a higher gene expression. The median values are expressed as horizontal lines, and the error bars represent interquartile range. CY, cystic; NC, non-cystic; HT, haploinsufficient; WT, wild-type; SC, severely cystic phenotype; CO, severely cystic phenotype controls. *Gapdh*, target expression normalized by *Gapdh*; *25*. *p<0.05 by Kruskal-Wallis with Dunn’s post-hoc test, followed by FDR correction.


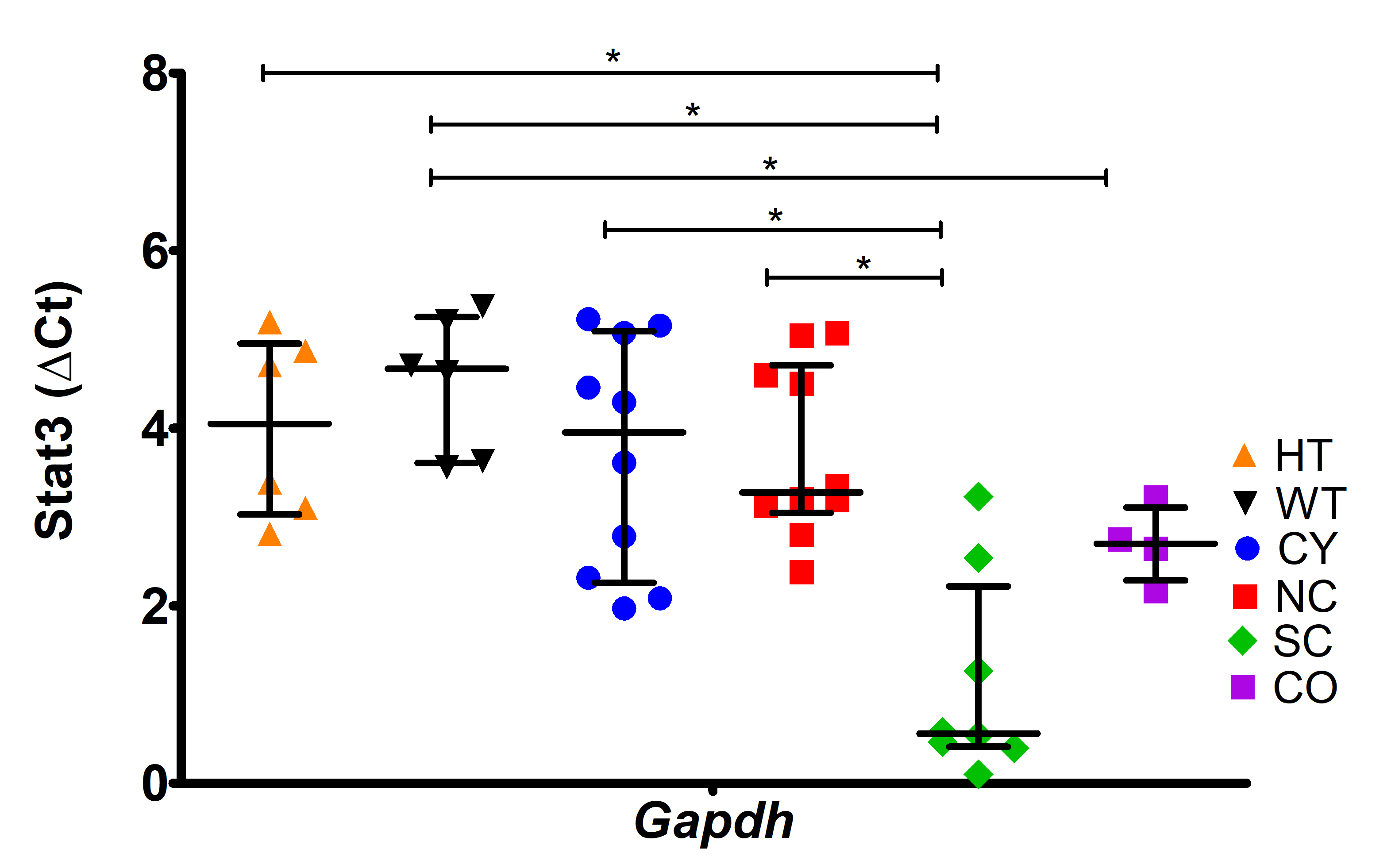


**Supplementary Figure S2.** The ΔCt values of *Stat3* target gene normalized by *Gapdh*. A lower ΔCt value indicates a higher gene expression. The median values are represented as horizontal lines, and the error bars represent interquartile range. CY, cystic; NC, non-cystic; HT, haploinsuficient; WT, wild-type; SC, severely cystic phenotype; CO, severely cystic phenotype controls. *p<0.05 by Kruskal-Wallis with Dunn’s post-hoc test, followed by FDR correction.


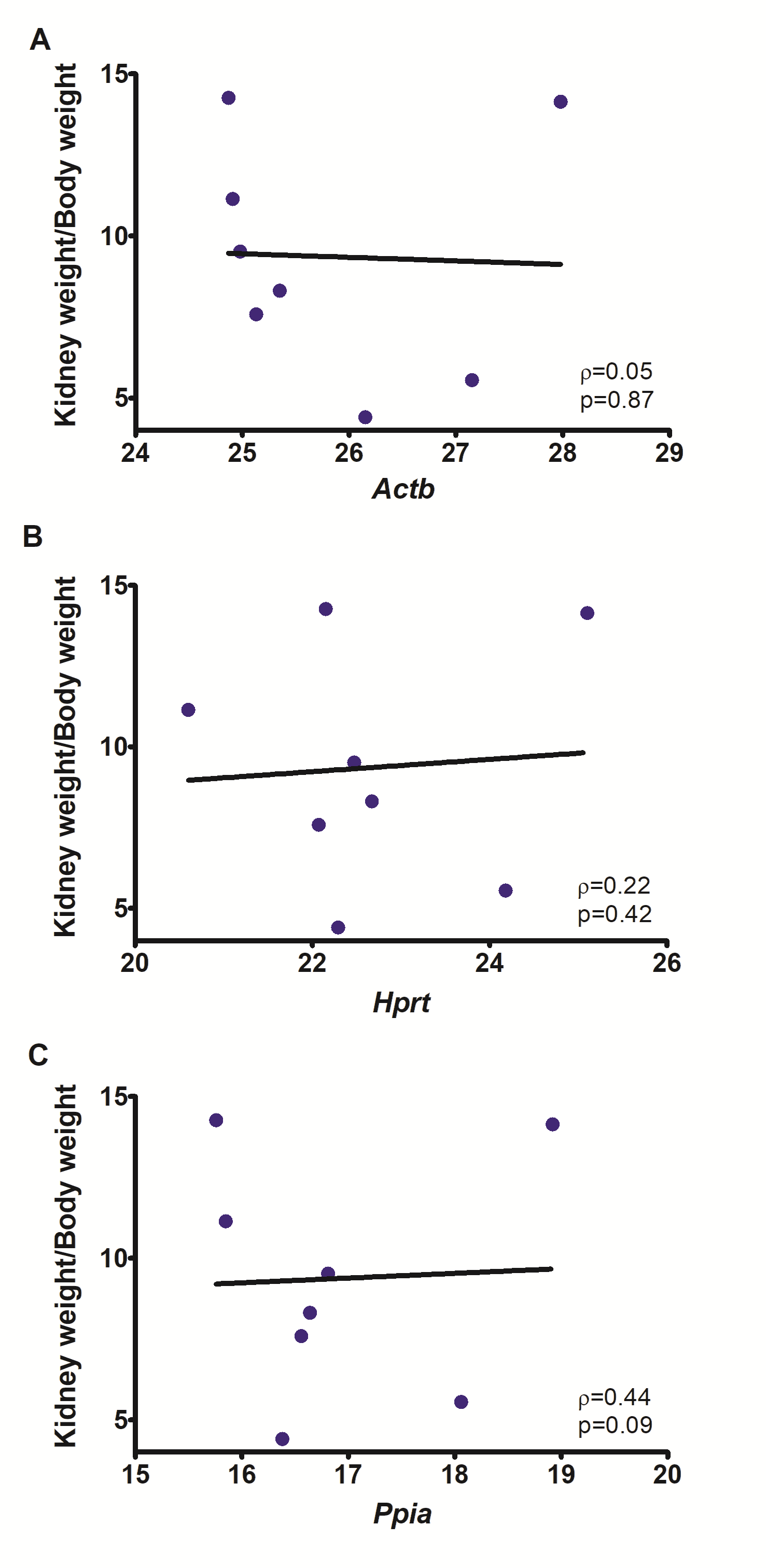


**Supplementary Figure S3**. Correlation matrix between the expression of *Actb* (**A**), *Hprt* (**B**), and *Ppia* (**C**) candidate housekeeping genes and the kidney weight (mg)/body weight (g) ratio in CY (cystic phenotype). *ρ*: Spearman’s rank correlation coefficient. *p<0.05.


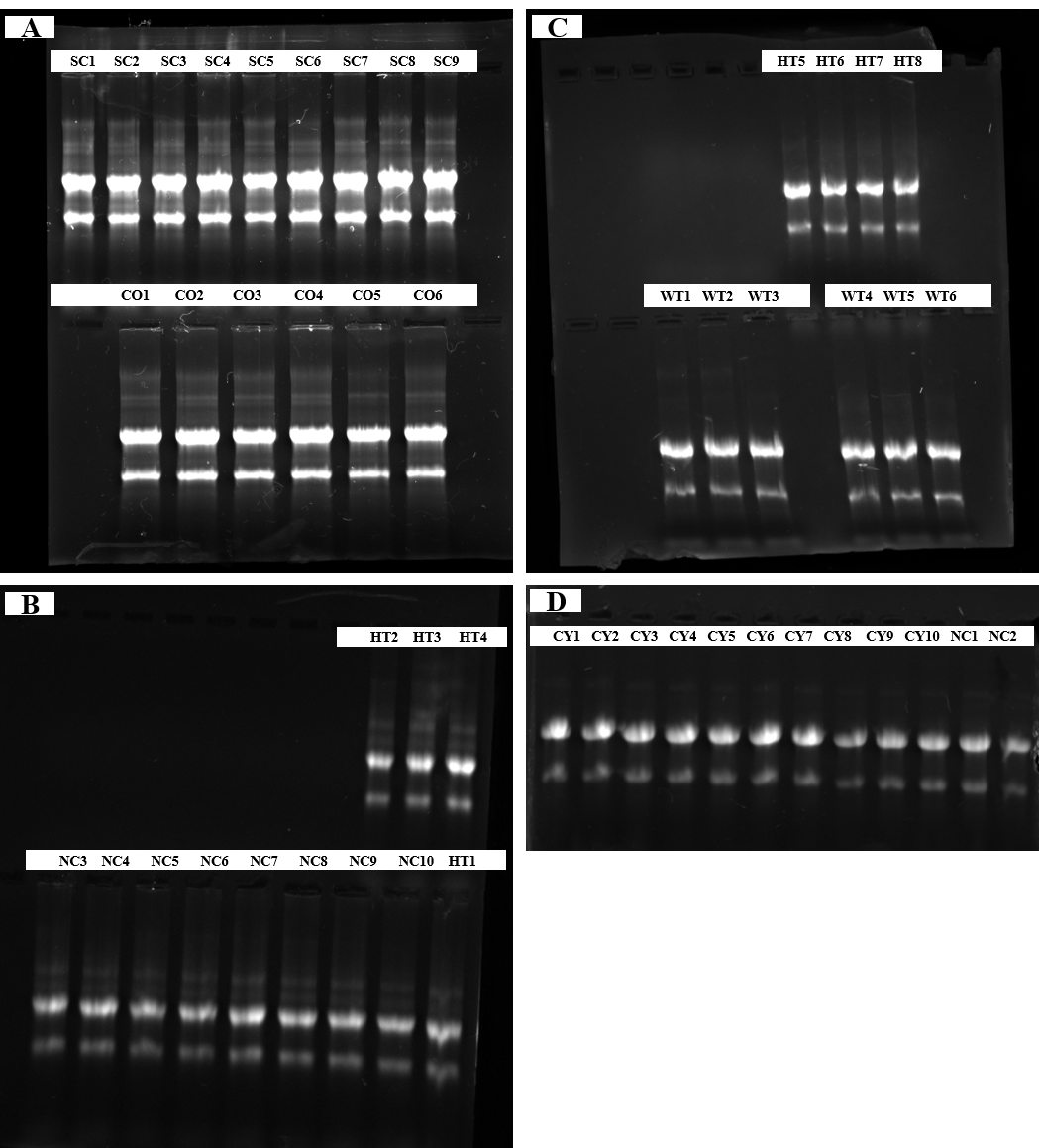


**Supplementary Figure S4**. Agarose gel electrophoresis images of RNA kidney tissues from mouse models orthologous to ADPKD. CY, cystic (1-10); NC, non-cystic (1-10); HT, haploinsufficient (1-8); WT, wild-type (1-6); SC, severe cystic phenotype (1-7); CO, severe cystic phenotype controls (1-5). The agarose gel electrophoresis images used in this figure were cropped from the original source gel images provided in the Supplementary Figures. Figure S5: SC (1-7) and CO (1-5) (**A**); Figure S6: NC (3-10) and HT (1-4) (**B**); Figure S7: HT (5-8) and WT (1-6) (**C**) and Figure S8: CY (1-10) and NC (1-2) (**D**). The cropped images were auto corrected for the same brightness/contrasts using PowerPoint image tools. Visible 28S (upper) and 18S (lower) rRNA bands were observed with high intensity in the gel images.


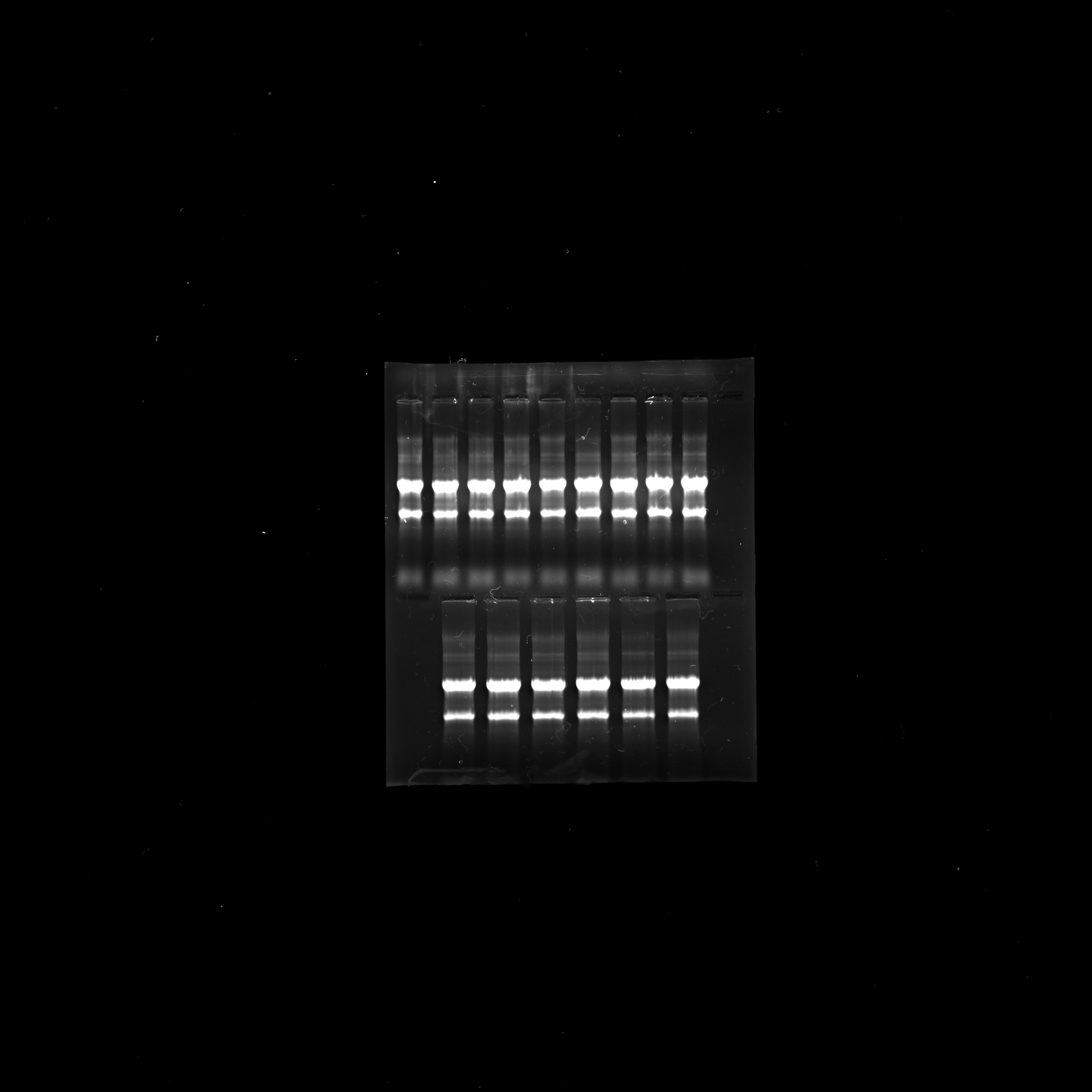


**Supplementary Figure S5**. Agarose gel electrophoresis images of RNA kidney tissues from mouse models orthologous to ADPKD. SC, severe cystic phenotype (1-7); CO, severe cystic phenotype controls (1-5). Visible 28S (upper) and 18S (lower) rRNA bands were observed with high intensity in the gel image. Original source gel image.


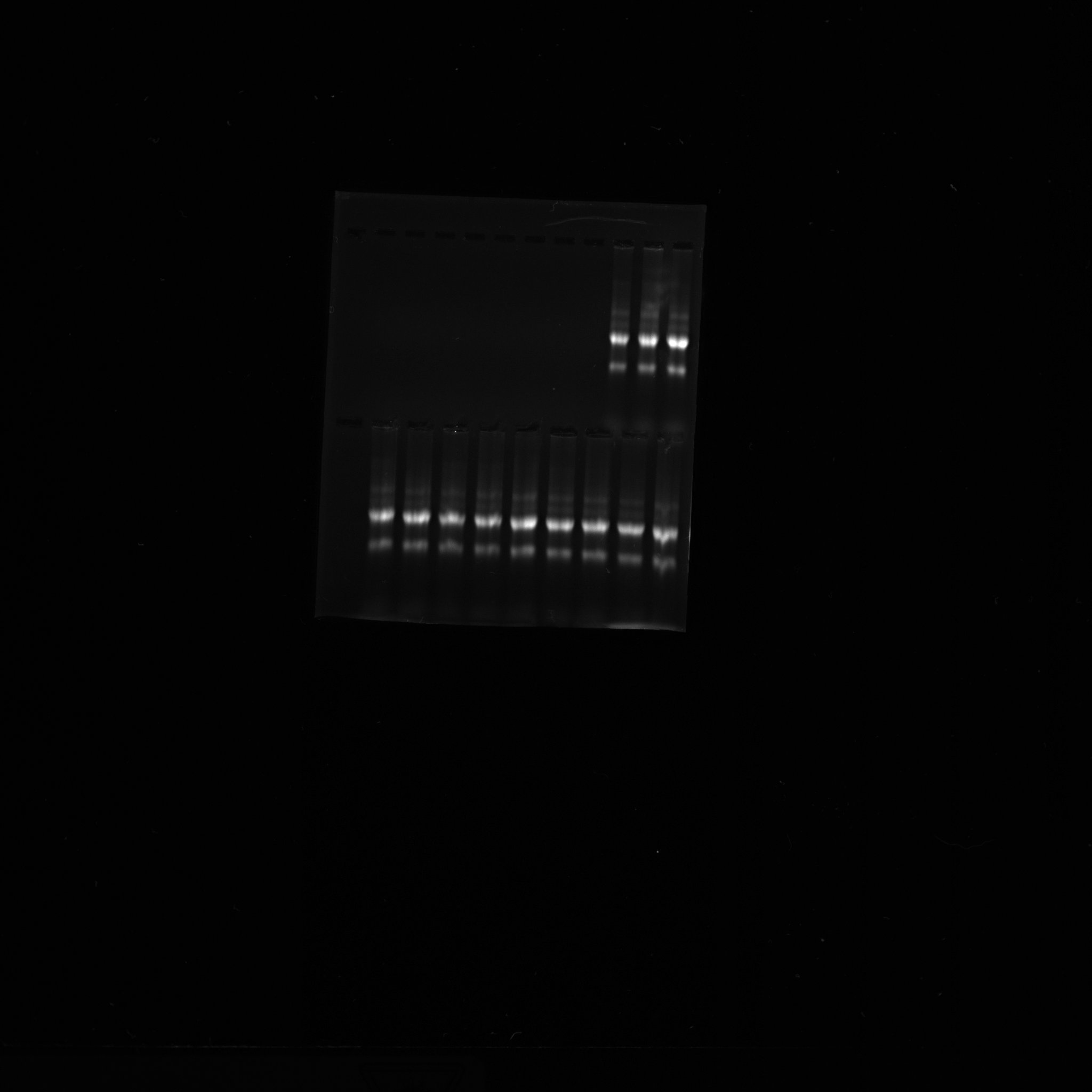


**Supplementary Figure S6**. Agarose gel electrophoresis images of RNA kidney tissues from mouse models orthologous to ADPKD. NC, non-cystic (3-10); HT, haploinsufficient (1-4). Visible 28S (upper) and 18S (lower) rRNA bands were observed with high intensity in the gel image. Original source gel image.


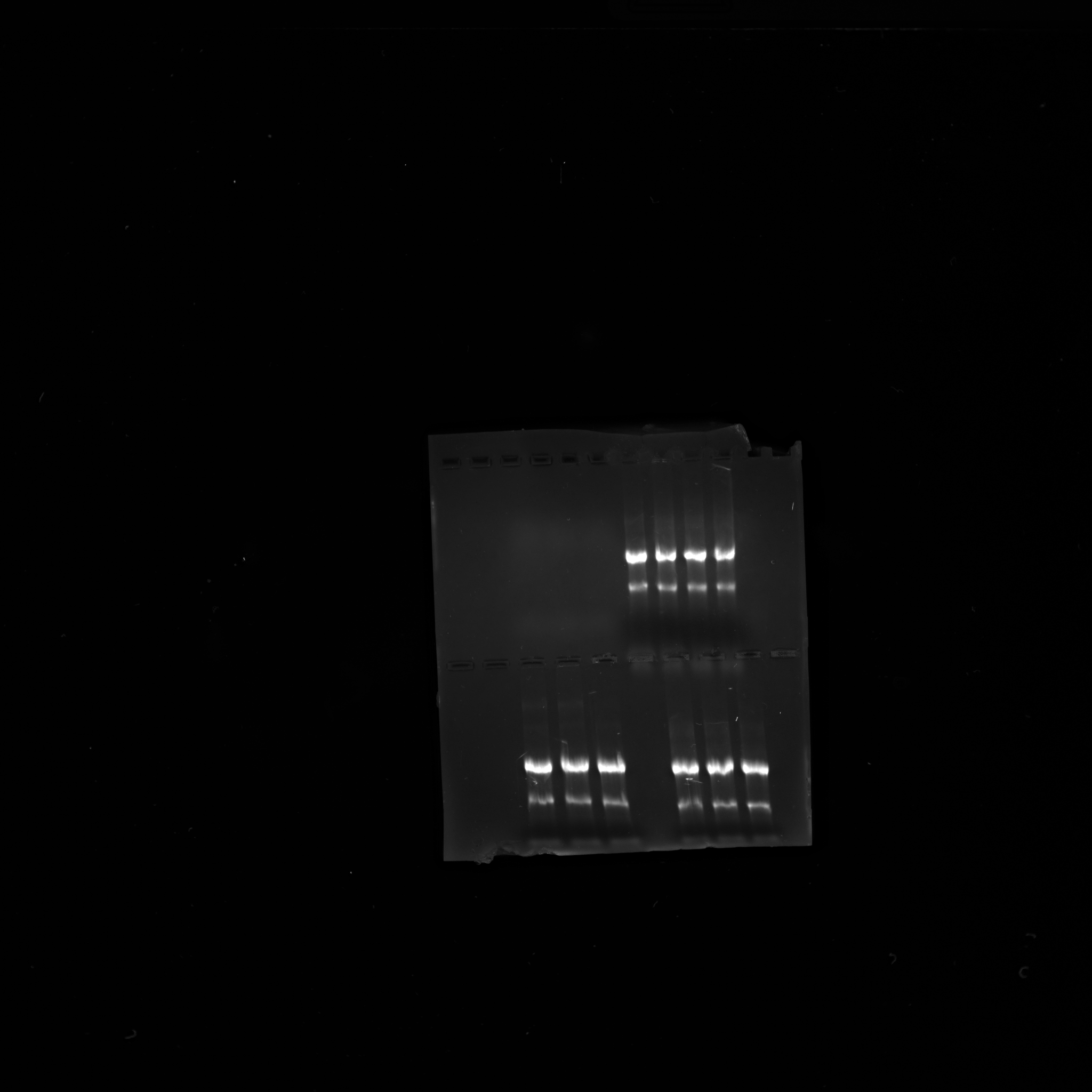


**Supplementary Figure S7**. Agarose gel electrophoresis images of RNA kidney tissues from mouse models orthologous to ADPKD. HT, haploinsufficient (5-8); WT, wild-type (1-6). Visible 28S (upper) and 18S (lower) rRNA bands were observed with high intensity in the gel image. Original source gel image.


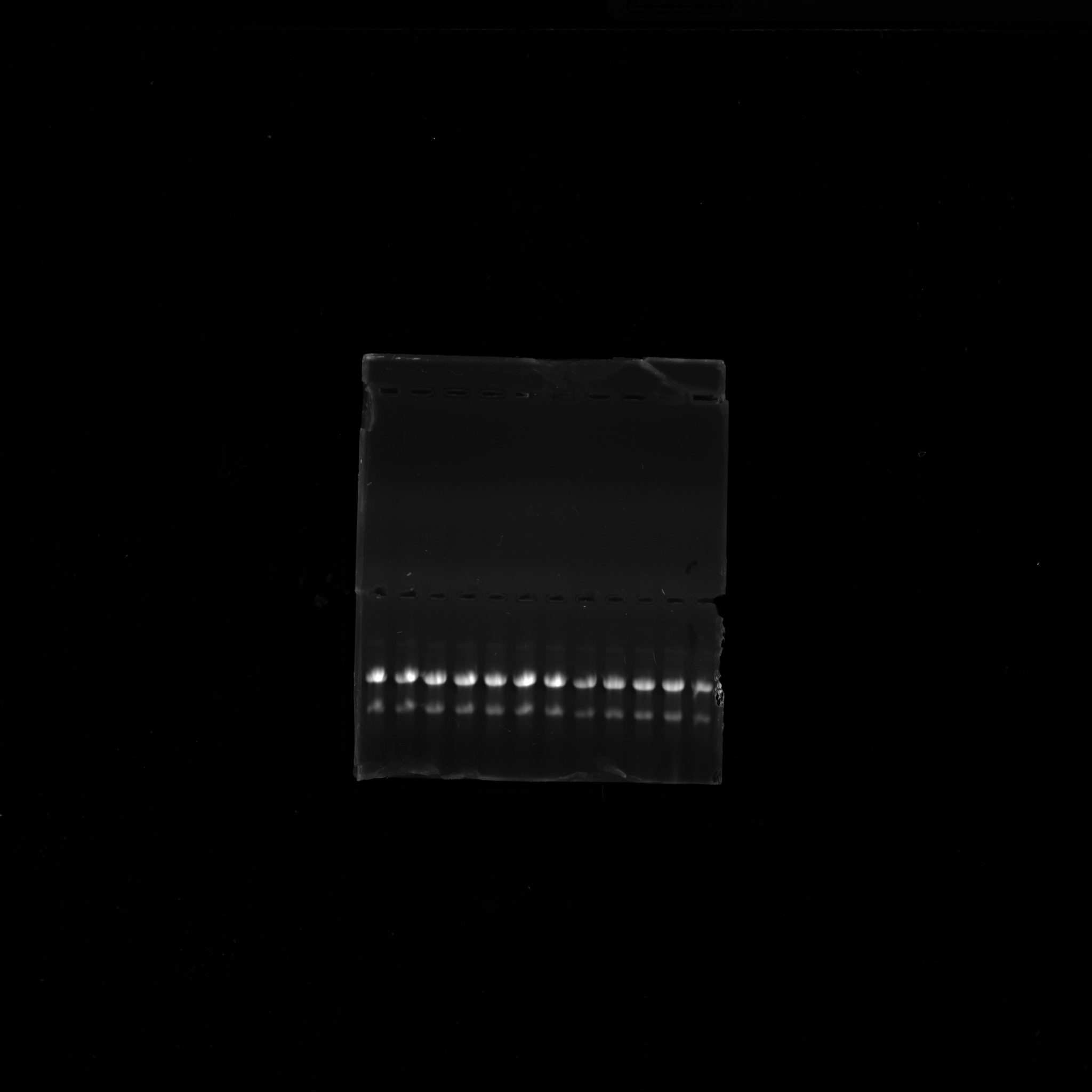


**Supplementary Figure S8**. Agarose gel electrophoresis images of RNA kidney tissues from mouse models orthologous to ADPKD. CY, cystic (1-10); NC, non-cystic (1-2). Visible 28S (upper) and 18S (lower) rRNA bands were observed with high intensity in the gel image. Original source gel image.

**Supplementary Table S1**. List of primers of the housekeeping genes.

| **Gene symbol** | **Sequence** | **Tm* (ºC)** |
| --- | --- | --- |
| **Actb** | D - 5' CGCAGCCACTGTCGAGT 3' | 60 |
|  | R - 5' GTCATCCATGGCGAACTGGT 3' |  |
| **Actg1** | D - 5 CACTAACTGGGACGACATGG 3' | 60 |
|  | R - 5 ACATTATCTGCGTCATCTTCTCT 3' |  |
| **B2m** | D - 5' ATACGCCTGCAGAGTTAAGC 3' | 60 |
|  | R - 5' TCACATGTCTCGATCCCAGT 3' |  |
| **Gapdh** | D - 5' TCAGGAGAGTGTTTCCTCGT 3' | 60 |
|  | R - 5' GCAACAATCTCCACTTTGCC 3' |  |
| **Hprt** | D - 5' TCATTATGCTGAGGATTTGGAAAG 3' | 60 |
|  | R - 5' GGCCTCCCATCTCCTTCATC 3' |  |
| **Pgam1** | D - 5' ATCAGCAAGGATCGCAGGTA 3' | 60 |
|  | R - 5' TTCATTCCAGAAGGGCAGTG 3' |  |
| **Ppia** | D - 5' CAGGTCCATCTACGGAGAGA 3' | 60 |
|  | R - 5' CATCCAGCCATTCAGTCTTG 3' |  |

*Melting Temperature (Tm).

**Supplementary Table S2.** Ranking of the seven candidate housekeeping genes determined by each of the six algorithms selected.

|  | **NormFinder** | **SD** | **GeNorm** | **M value** | **RefFinder** | **Geomean** | **ΔCt method** | **Mean SD** | **Bestkeeper** | **CV** | **SD** | **DataAssist** | **Score** |
| --- | --- | --- | --- | --- | --- | --- | --- | --- | --- | --- | --- | --- | --- |
| **All** | *Ppia* | 0,22 | *Actg1* | 0,33 | *Ppia* | 1,00 | *Ppia* | 0,80 | *Hprt* | 2,67 | 0,60 | *Ppia* | 0,68 |
|  | *Hprt* | 0,30 | *Actb* | 0,33 | *Hprt* | 1,68 | *Hprt* | 0,86 | *Actb* | 3,10 | 0,79 | *Hprt* | 0,73 |
|  | *Pgam1* | 0,41 | *Ppia* | 0,53 | *Pgam1* | 3,87 | *Actg1* | 0,91 | *Ppia* | 3,24 | 0,54 | *Actg1* | 0,81 |
|  | *B2m* | 0,49 | *Hprt* | 0,55 | *Actg1* | 4,05 | *Actb* | 0,92 | *Pgam1* | 3,50 | 0,69 | *Actb* | 0,81 |
|  | *Actb* | 0,53 | *Pgam1* | 0,62 | *Actb* | 4,23 | *Pgam1* | 0,94 | *B2m* | 3,51 | 0,63 | *Pgam1* | 0,83 |
|  | *Actg1* | 0,56 | *B2m* | 0,70 | *B2m* | 5,42 | *B2m* | 1,05 | *Gapdh* | 4,32 | 0,82 | *B2m* | 0,95 |
|  | *Gapdh* | 0,83 | *Gapdh* | 0,85 | *Gapdh* | 7,00 | *Gapdh* | 1,38 | *Actg1* | 4,77 | 0,80 | *Gapdh* | 1,27 |
| **CY** | *Ppia* | 0,09 | *Ppia* | 0,18 | *Ppia* | 1,41 | *Ppia* | 0,63 | *Actb* | 3,25 | 0,84 | *Ppia* | 0,50 |
|  | *Pgam1* | 0,09 | *Actg1* | 0,18 | *Actg1* | 2,45 | *Actg1* | 0,66 | *B2m* | 3,35 | 0,60 | *Pgam1* | 0,54 |
|  | *Actg1* | 0,20 | *Pgam1* | 0,19 | *Pgam1* | 2,91 | *Pgam1* | 0,70 | *Gapdh* | 3,72 | 0,70 | *Actg1* | 0,55 |
|  | *Actb* | 0,36 | *Actb* | 0,28 | *Gapdh* | 4,30 | *Actb* | 0,78 | *Hprt* | 3,87 | 0,88 | *B2m* | 0,61 |
|  | *B2m* | 0,37 | *B2m* | 0,34 | *Hprt* | 4,40 | *Hprt* | 0,79 | *Pgam1* | 3,92 | 0,76 | *Hprt* | 0,65 |
|  | *Hprt* | 0,50 | *Hprt* | 0,39 | *B2m* | 4,56 | *B2m* | 0,84 | *Ppia* | 4,15 | 0,70 | *Actb* | 0,66 |
|  | *Gapdh* | 1,21 | *Gapdh* | 0,61 | *Actb* | 4,86 | *Gapdh* | 1,30 | *Actg1* | 4,53 | 0,77 | *Gapdh* | 1,21 |
| **NC** | *Pgam1* | 0,09 | *Pgam1* | 0,19 | *Hprt* | 1,50 | *Hprt* | 0,52 | *Actb* | 1,75 | 0,45 | *Ppia* | 0,46 |
|  | *Ppia* | 0,14 | *Actb* | 0,19 | *Actb* | 1,86 | *Actb* | 0,54 | *Actg1* | 2,02 | 0,34 | *Pgam1* | 0,50 |
|  | *Actb* | 0,15 | *Hprt* | 0,26 | *Ppia* | 2,45 | *Ppia* | 0,56 | *Hprt* | 2,05 | 0,45 | *Hprt* | 0,50 |
|  | *Hprt* | 0,28 | *Actg1* | 0,29 | *Pgam1* | 3,72 | *Pgam1* | 0,57 | *Pgam1* | 2,32 | 0,44 | *Actb* | 0,50 |
|  | *Actg1* | 0,28 | *Ppia* | 0,31 | *Actg1* | 3,98 | *Actg1* | 0,60 | *Ppia* | 2,40 | 0,40 | *Actg1* | 0,51 |
|  | *B2m* | 0,43 | *B2m* | 0,35 | *B2m* | 6,00 | *B2m* | 0,63 | *B2m* | 2,83 | 0,51 | *B2m* | 0,56 |
|  | *Gapdh* | 0,95 | *Gapdh* | 0,51 | *Gapdh* | 7,00 | *Gapdh* | 0,96 | *Gapdh* | 4,88 | 0,92 | *Gapdh* | 0,95 |
| **HT** | *Pgam1* | 0,12 | *Ppia* | 0,25 | *Pgam1* | 1,41 | *Pgam1* | 0,64 | *Hprt* | 2,13 | 0,48 | *Hprt* | 0,57 |
|  | *Hprt* | 0,15 | *Hprt* | 0,25 | *Actg1* | 2,11 | *Actg1* | 0,64 | *Ppia* | 2,75 | 0,46 | *B2m* | 0,60 |
|  | *Actg1* | 0,36 | *B2m* | 0,27 | *Hprt* | 2,83 | *B2m* | 0,67 | *Actb* | 2,87 | 0,76 | *Pgam1* | 0,62 |
|  | *B2m* | 0,37 | *Pgam1* | 0,37 | *B2m* | 3,00 | *Hprt* | 0,74 | *Pgam1* | 2,87 | 0,57 | *Ppia* | 0,65 |
|  | *Actb* | 0,40 | *Actg1* | 0,42 | *Ppia* | 4,16 | *Ppia* | 0,90 | *Gapdh* | 3,14 | 0,60 | *Actg1* | 0,73 |
|  | *Ppia* | 0,49 | *Actb* | 0,45 | *Actb* | 5,73 | *Actb* | 0,92 | *B2m* | 3,52 | 0,65 | *Actb* | 0,78 |
|  | *Gapdh* | 1,01 | *Gapdh* | 0,61 | *Gapdh* | 7,00 | *Gapdh* | 1,42 | *Actg1* | 3,97 | 0,70 | *Gapdh* | 1,04 |
| **WT** | *Ppia* | 0,10 | *B2m* | 0,21 | *Pgam1* | 1,19 | *Pgam1* | 0,50 | *Hprt* | 1,56 | 0,35 | *Ppia* | 0,48 |
|  | *Pgam1* | 0,11 | *Pgam1* | 0,21 | *Actb* | 1,86 | *Actb* | 0,50 | *Actb* | 1,56 | 0,41 | *Pgam1* | 0,49 |
|  | *Hprt* | 0,18 | *Ppia* | 0,23 | *Ppia* | 3,46 | *Ppia* | 0,62 | *Pgam1* | 2,29 | 0,45 | *B2m* | 0,50 |
|  | *B2m* | 0,21 | *Hprt* | 0,27 | *Hprt* | 3,46 | *Actg1* | 0,63 | *Ppia* | 2,41 | 0,40 | *Hprt* | 0,55 |
|  | *Actb* | 0,25 | *Actb* | 0,31 | *Actg1* | 4,53 | *B2m* | 0,66 | *Gapdh* | 2,52 | 0,46 | *Actb* | 0,65 |
|  | *Actg1* | 0,50 | *Actg1* | 0,35 | *B2m* | 5,48 | *Hprt* | 0,66 | *B2m* | 2,70 | 0,50 | *Actg1* | 0,73 |
|  | *Gapdh* | 0,96 | *Gapdh* | 0,51 | *Gapdh* | 6,44 | *Gapdh* | 1,08 | *Actg1* | 4,17 | 0,73 | *Gapdh* | 1,04 |
| **SC** | *Ppia* | 0,15 | *Hprt* | 0,15 | *Ppia* | 1,68 | *Ppia* | 0,59 | *Gapdh* | 2,50 | 0,51 | *Ppia* | 0,57 |
|  | *Actg1* | 0,29 | *Actg1* | 0,15 | *Actb* | 1,78 | *Actb* | 0,59 | *Actb* | 3,81 | 0,95 | *Actg1* | 0,57 |
|  | *Actb* | 0,37 | *Actb* | 0,19 | *Pgam1* | 3,40 | *Pgam1* | 0,76 | *Hprt* | 4,63 | 1,04 | *Actb* | 0,60 |
|  | *Hprt* | 0,44 | *Pgam1* | 0,24 | *Gapdh* | 3,83 | *Actg1* | 0,77 | *Ppia* | 4,88 | 0,83 | *Hprt* | 0,63 |
|  | *Pgam1* | 0,51 | *Ppia* | 0,29 | *Actg1* | 4,43 | *Hprt* | 0,80 | *Pgam1* | 4,98 | 1,03 | *Pgam1* | 0,72 |
|  | *Gapdh* | 0,76 | *Gapdh* | 0,49 | *Hprt* | 4,79 | *Gapdh* | 0,91 | *B2m* | 5,38 | 0,93 | *Gapdh* | 1,01 |
|  | *B2m* | 1,35 | *B2m* | 0,73 | *B2m* | 5,12 | *B2m* | 1,31 | *Actg1* | 6,13 | 1,00 | *B2m* | 1,37 |
| **CO** | *Pgam1* | 0,08 | *Ppia* | 0,14 | *Pgam1* | 1,19 | *Pgam1* | 0,39 | *Hprt* | 0,77 | 0,17 | *Actg1* | 0,32 |
|  | *Actg1* | 0,09 | *Actg1* | 0,14 | *Gapdh* | 2,08 | *Actg1* | 0,39 | *Gapdh* | 0,77 | 0,15 | *Pgam1* | 0,33 |
|  | *Ppia* | 0,11 | *Pgam1* | 0,15 | *Actg1* | 2,29 | *Gapdh* | 0,39 | *Pgam1* | 0,91 | 0,18 | *Ppia* | 0,34 |
|  | *Hprt* | 0,14 | *Hprt* | 0,17 | *Hprt* | 4,47 | *Hprt* | 0,62 | *Actb* | 1,11 | 0,28 | *Hprt* | 0,35 |
|  | *Gapdh* | 0,30 | *Actb* | 0,20 | *Ppia* | 4,47 | *Ppia* | 0,62 | *Actg1* | 1,24 | 0,20 | *Actb* | 0,44 |
|  | *Actb* | 0,33 | *Gapdh* | 0,24 | *Actb* | 6,00 | *Actb* | 0,66 | *Ppia* | 1,50 | 0,24 | *Gapdh* | 0,45 |
|  | *B2m* | 1,01 | *B2m* | 0,43 | *B2m* | 7,00 | *B2m* | 1,03 | *B2m* | 4,22 | 0,75 | *B2m* | 0,92 |
| **CY+NC** | *Pgam1* | 0,04 | *Ppia* | 0,21 | *Ppia* | 1,00 | *Ppia* | 0,59 | *Actb* | 2,61 | 0,67 | *Ppia* | 0,47 |
|  | *Ppia* | 0,05 | *Actg1* | 0,21 | *Actg1* | 2,28 | *Pgam1* | 0,63 | *Hprt* | 2,94 | 0,66 | *Pgam1* | 0,49 |
|  | *Actg1* | 0,08 | *Pgam1* | 0,25 | *Pgam1* | 2,38 | *Actg1* | 0,63 | *B2m* | 3,09 | 0,55 | *Actg1* | 0,51 |
|  | *Actb* | 0,10 | *Actb* | 0,29 | *Actb* | 3,72 | *Actb* | 0,67 | *Pgam1* | 3,12 | 0,60 | *Actb* | 0,56 |
|  | *B2m* | 0,14 | *Hprt* | 0,34 | *Hprt* | 5,00 | *Hprt* | 0,69 | *Ppia* | 3,25 | 0,54 | *Hprt* | 0,60 |
|  | *Hprt* | 0,14 | *B2m* | 0,39 | *B2m* | 6,00 | *B2m* | 0,73 | *Actg1* | 3,35 | 0,56 | *B2m* | 0,60 |
|  | *Gapdh* | 0,34 | *Gapdh* | 0,57 | *Gapdh* | 7,00 | *Gapdh* | 1,12 | *Gapdh* | 4,31 | 0,81 | *Gapdh* | 1,09 |
| **HT+WT** | *Pgam1* | 0,05 | *Ppia* | 0,23 | *Actg1* | 2,06 | *Actg1* | 0,69 | *Hprt* | 1,89 | 0,42 | *Hprt* | 0,52 |
|  | *Hprt* | 0,07 | *Hprt* | 0,23 | *B2m* | 2,06 | *B2m* | 0,70 | *Actb* | 2,25 | 0,59 | *B2m* | 0,54 |
|  | *B2m* | 0,12 | *B2m* | 0,27 | *Hprt* | 2,34 | *Hprt* | 0,71 | *Ppia* | 2,64 | 0,44 | *Pgam1* | 0,55 |
|  | *Ppia* | 0,12 | *Pgam1* | 0,34 | *Pgam1* | 2,83 | *Pgam1* | 0,73 | *Pgam1* | 2,64 | 0,52 | *Ppia* | 0,55 |
|  | *Actb* | 0,13 | *Actg1* | 0,39 | *Ppia* | 3,94 | *Ppia* | 0,77 | *B2m* | 3,16 | 0,58 | *Actb* | 0,65 |
|  | *Actg1* | 0,17 | *Actb* | 0,41 | *Actb* | 5,48 | *Actb* | 0,78 | *Gapdh* | 3,39 | 0,63 | *Actg1* | 0,66 |
|  | *Gapdh* | 0,40 | *Gapdh* | 0,57 | *Gapdh* | 7,00 | *Gapdh* | 1,25 | *Actg1* | 4,17 | 0,73 | *Gapdh* | 1,03 |
| **SC+CO** | *Ppia* | 0,13 | *Hprt* | 0,18 | *Ppia* | 1,00 | *Ppia* | 0,68 | *Actb* | 2,69 | 0,67 | *Ppia* | 0,55 |
|  | *Actg1* | 0,14 | *Actg1* | 0,18 | *Actb* | 2,21 | *Actg1* | 0,73 | *Hprt* | 3,01 | 0,67 | *Actg1* | 0,55 |
|  | *Hprt* | 0,26 | *Actb* | 0,25 | *Actg1* | 3,31 | *Actb* | 0,75 | *Gapdh* | 3,26 | 0,65 | *Hprt* | 0,60 |
|  | *Actb* | 0,40 | *Ppia* | 0,29 | *Hprt* | 4,61 | *Pgam1* | 0,78 | *Ppia* | 3,73 | 0,62 | *Actb* | 0,65 |
|  | *Pgam1* | 0,50 | *Pgam1* | 0,40 | *Pgam1* | 4,86 | *Hprt* | 0,80 | *Actg1* | 4,18 | 0,67 | *Pgam1* | 0,76 |
|  | *Gapdh* | 0,52 | *Gapdh* | 0,54 | *Gapdh* | 5,05 | *Gapdh* | 0,88 | *Pgam1* | 4,33 | 0,87 | *Gapdh* | 0,91 |
|  | *B2m* | 0,70 | *B2m* | 0,74 | *B2m* | 5,12 | *B2m* | 1,38 | *B2m* | 4,79 | 0,84 | *B2m* | 1,29 |

All, all samples; CY, cystic; NC, non-cystic; HT, haploinsuficient; WT, wild-type; SC, severe cystic phenotype; CO, severe cystic phenotype controls. NormFinder (version 0.953; https://moma.dk/normfinder-software), GeNorm (version 2.2; https://genorm.cmgg.be/), BestKeeper (version 1.0; https://www.gene-quantification.de/bestkeeper.html), DataAssist (version 3.01; https://www.thermofisher.com/br/en/home/technical-resources/software-downloads/dataassist-software.html), the comparative ΔCt method and RefFinder (https://www.heartcure.com.au/reffinder/).

Genes in bold: higher frequency of appearance in softwares.

**Supplementary Table S3.** Ranking of the seven candidate housekeeping genes determined by each of the six algorithms selected (using groups CY and NC considering all samples and the analysis considering only samples CY and NC without deletion).

|  | **NormFinder** | **SD** | **RefFinder** | **Geomean** | **ΔCt method** | **Mean SD** | **Bestkeeper** | **CV** | **SD** | **DataAssist** | **Score** |
| --- | --- | --- | --- | --- | --- | --- | --- | --- | --- | --- | --- |
| **CY+NC** | *Pgam1* | 0,04 | *ppia* | 1,00 | *ppia* | 0,59 | *actb* | 2,61 | 0,67 | *ppia* | 0,47 |
|  | *ppia* | 0,05 | *actg1* | 2,28 | *pgam1* | 0,63 | *hprt* | 2,94 | 0,66 | *pgam1* | 0,49 |
|  | *actg1* | 0,08 | *pgam1* | 2,38 | *actg1* | 0,63 | *b2m* | 3,09 | 0,55 | *actg1* | 0,51 |
|  | *actb* | 0,10 | *actb* | 3,72 | *actb* | 0,67 | *pgam1* | 3,12 | 0,60 | *actb* | 0,56 |
|  | *b2m* | 0,14 | *hprt* | 5,00 | *hprt* | 0,69 | *ppia* | 3,25 | 0,54 | *hprt* | 0,60 |
|  | *hprt* | 0,14 | *b2m* | 6,00 | *b2m* | 0,73 | *actg1* | 3,35 | 0,56 | *b2m* | 0,60 |
|  | *gapdh* | 0,34 | *gapdh* | 7,00 | *gapdh* | 1,12 | *gapdh* | 4,31 | 0,81 | *gapdh* | 1,09 |
| **CY+NC without deletion** | *pgam1* | 0,152 | *ppia* | 1,41 | *ppia* | 0,59 | *actb* | 2,128887 | 0,541001 | *ppia* | 0,475 |
|  | *ppia* | 0,160 | *actg1* | 1,73 | *pgam1* | 0,61 | *hprt* | 2,133858 | 0,472996 | *pgam1* | 0,521 |
|  | *actg1* | 0,261 | *pgam1* | 2,21 | *actg1* | 0,63 | *ppia* | 2,256199 | 0,371576 | *actg1* | 0,534 |
|  | *actb* | 0,335 | *hprt* | 4,73 | *hprt* | 0,7 | *actg1* | 2,324485 | 0,384991 | *b2m* | 0,563 |
|  | *b2m* | 0,368 | *actb* | 4,86 | *actb* | 0,71 | *pgam1* | 2,437279 | 0,462459 | *hprt* | 0,582 |
|  | *hprt* | 0,424 | *b2m* | 5,05 | *b2m* | 0,8 | *b2m* | 2,569865 | 0,4521 | *actb* | 0,613 |
|  | *gapdh* | 1,023 | *gapdh* | 6,74 | *gapdh* | 0,98 | *gapdh* | 3,81332 | 0,708502 | *gapdh* | 1,031 |

CY + NC: all samples (*Pkd1*^flox/flox^:*Nestin*^cre+^, *Pkd1*^flox/-^:*Nestin*^cre+^ *, Pkd1*^flox/flox^:*Nestin*^cre-^ and *Pkd1*^flox/-^:*Nestin*^cre-^ )

CY + NC without deletion: (*Pkd1*^flox/flox^:*Nestin*^cre+^ and *Pkd1*^flox/flox^:*Nestin*^cre-^ ).

NormFinder (version 0.953; https://moma.dk/normfinder-software), GeNorm (version 2.2; https://genorm.cmgg.be/), BestKeeper (version 1.0; https://www.gene-quantification.de/bestkeeper.html), DataAssist (version 3.01; https://www.thermofisher.com/br/en/home/technical-resources/software-downloads/dataassist-software.html), the comparative ΔCt method and RefFinder (https://www.heartcure.com.au/reffinder/).

Genes in bold: higher frequency of appearance in softwares.

**Supplementary Table S4.** RNA Integrity analyzed by Agilent 2100 Bioanalyzer 6000 Nanochip using kidney samples (CY= 10, NC = 10, HT = 6, WT = 6, SC = 7 and CO = 5)

| **Sample** | **RIN** |
| --- | --- |
| **CY1** | 7.0 |
| **CY2** | 6.0 |
| **CY3** | 8.0 |
| **CY4** | 8.0 |
| **CY5** | 8.1 |
| **CY6** | 8.0 |
| **CY7** | 8.0 |
| **CY8** | 7.0 |
| **CY9** | 7.0 |
| **CY10** | 5.9 |
| **NC1** | 7.0 |
| **NC2** | 8.0 |
| **NC3** | 6.0 |
| **NC4** | 7.0 |
| **NC5** | 6.1 |
| **NC6** | 8.0 |
| **NC7** | 8.0 |
| **NC8** | 8.0 |
| **NC9** | 7.0 |
| **NC10** | 5,9 |
| **HT1** | 8,7 |
| **HT2** | 8.0 |
| **HT3** | 7.0 |
| **HT4** | 8.0 |
| **HT5** | 8.1 |
| **HT6** | 7.0 |
| **WT1** | 7.0 |
| **WT2** | 8.0 |
| **WT3** | 8.0 |
| **WT4** | 7.8 |
| **WT5** | 6.9 |
| **WT6** | 8.9 |
| **SC1** | 8.9 |
| **SC2** | 7.0 |
| **SC3** | 8.0 |
| **SC4** | 7.8 |
| **SC5** | 8.8 |
| **SC6** | 8.0 |
| **SC7** | 7.0 |
| **CO1** | 8.0 |
| **CO2** | 8.1 |
| **CO3** | 7.0 |
| **CO4** | 8.0 |
| **CO5** | 7.0 |

RIN: RNA Integrity Number

**Competing Interests:** The authors declare no competing interests.
